# Engaging athletes as research participants. A document analysis of published sport science literature

**DOI:** 10.1002/ejsc.12198

**Published:** 2024-09-17

**Authors:** Lachlan Mitchell, Josie Ratcliff, Louise M. Burke, Adrienne Forsyth

**Affiliations:** ^1^ School of Behavioural and Health Sciences Australian Catholic University North Sydney New South Wales Australia; ^2^ Mary MacKillop Institute for Health Research Australian Catholic University Melbourne Victoria Australia; ^3^ School of Behavioural and Health Sciences Australian Catholic University Melbourne Victoria Australia

**Keywords:** athlete engagement, collaboration, embedded researcher, gatekeeper, research co‐design

## Abstract

Sport science practitioners utilise findings from peer reviewed research to inform practice. Fewer studies are conducted with high performance athletes, however, than those involving recreationally active participants. Noting that research findings from recreational athletes may not be generalisable to the elite, there is a need to engage the latter cohort in research with better potential to influence health and performance. This study identified methods used to engage and recruit highly trained, elite and world class athletes as research participants. A document analysis was conducted using a purposive sample of peer‐reviewed sport science literature. All articles published in 2022 from 18 highly ranked sport science journals were screened for inclusion. Studies investigating athletes ranked as highly trained/national level or above were included. All details related to participant recruitment were extracted from included articles, with the content being coded and thematically analysed using an interpretivist approach. A total of 439 studies from the 2356 screened were included in the analysis. Five primary themes of recruitment strategies were identified, beneath an overarching strategy of purposeful, convenience sampling. Recruitment themes related to the use of a gatekeeper, the research environment providing convenient access to athletes, promoting the study electronically, utilising professional networks and recruiting at training or competition. Engaging athletes through a gatekeeper is a prominent strategy to involve elite athletes in research. It is suggested that researchers work collaboratively with team or organisation personnel to promote recruitment, creating co‐designed approaches that address issues most relevant to athletes and staff.

## Highlights

- Engaging elite athletes through a gatekeeper appears to be a successful strategy for participant recruitment.
- Embedding a researcher within the sporting organisation or team, or collaborating with team personnel, are two possible approaches to using a gatekeeper and will likely promote recruitment as well as the co‐design of sport science research.
- Taking advantage of the university environment where student‐athletes are available is a convenient and potentially effective approach to engaging athletes for recruitment.

## 1 INTRODUCTION

Evidence‐based practice is a fundamental principle that guides exercise and nutrition recommendations (Coutts, 2017; Fullagar et al., 2019). In a sporting context, evidence‐based practice has been described as ‘the integration of coaching expertise, athlete values and the best relevant research evidence into the decision‐making process for day‐to‐day service delivery to athletes’ (Coutts, 2017). Observational research evidence provides a description of a population or outcome, while experimental studies relate the cause and effect of an exercise or nutrition intervention (Burke et al., 2018). Practitioners utilise findings from peer reviewed research to inform their practice and research questions are often generated and evolve from observations of real‐world behaviours, events and outcomes (Hawley et al., 2018). Given the importance of evidence‐based practice in exercise and nutrition, addressing research questions in sport science has the potential to improve health, performance and recovery of athletes and the wider population (Fullagar et al., 2019). Research in sport science is therefore necessary to support practitioners and athletes (Bishop et al., 2006).

The generalisability of research outcomes depends in part on characteristics of the study from which outcomes are derived. Study findings should only be applied to populations and scenarios that reflect those of the study itself (Betts et al., 2020). While a homogenous participant sample may improve internal validity, it likely renders findings relevant only to populations similar to that from which the sample was drawn. In the context of sport science research, caution should be used when applying findings obtained from research with untrained or recreationally active individuals to highly trained, elite and world class athletes (Burke et al., 2018). There are many examples in sport science research where adaptations or performance improvements with trained athletes following an intervention are not replicated with highly trained or elite athletes. For example, in moderately trained athletes, changes in endurance performance following a training block with periodised carbohydrate availability were greater than those of a control group whose diet provided continuous high carbohydrate availability (Marquet et al., 2016). Meanwhile, no such advantage was reported when elite athletes followed equivalent interventions (Burke et al., 2017). A similar trend in outcomes has been observed following dietary nitrate consumption, with a negative correlation between performance improvement following supplementation and participant fitness level (Porcelli et al., 2015).

Given the limitations of transferring research findings with active and trained individuals to higher calibre athletes, it is pertinent to involve highly trained and elite cohorts in the investigations which target their unique characteristics and needs. Participant recruitment is a general research challenge, having been described in populations as diverse as university students, pregnant women and cancer patients (Frew et al., 2014; Sygna et al., 2015; Vadeboncoeur et al., 2017). Nevertheless, conducting research with highly trained and elite athletes has novel challenges, including the reluctance of athletes and their coach staff to accept disruptions to their training and competition preparation (Coutts, 2017). Athletes and teams may also be hesitant to disclose information that could provide a competitive advantage to others. Furthermore, by definition the populations of highly trained, elite and world class athletes become increasingly smaller than their active and moderately trained counterparts, creating great logistical challenges around study recruitment (McKay et al., 2022). Unfortunately, strategies that can successfully address these challenges have not been thoroughly investigated. Such information has the potential to positively influence athlete health and performance through increasing the scope, quality and impact of sports science research. Therefore, the aim of this study was to identify methods used to engage and recruit highly trained, elite and world class athletes as participants into research from a purposeful sample of sports science literature.

## 2 METHODS

### 2.1 Procedure

A qualitative approach using document analysis of sports science literature was conducted. Document analysis provides the researcher with scope to uncover meaning, develop understanding and discover insights relevant to the research problem (Bowen, 2009). A constructionist epistemological position was adopted, whereby knowledge was constructed through engagement with and interpretation of document content (Moon et al., 2014). Reporting of results was guided by the Checklist for Assessment and Reporting of Document Analysis (Cleland et al., 2023). The analysis was conducted according to the methods outlined by Bowen; this involved finding, selecting, appraising and synthesising data contained within documents (Bowen, 2009). A purposeful sample of peer reviewed sports science articles were selected for analysis. All articles published in the year 2022 in the top 15 ranked journals of the Sport Science category based on impact factor according to Scimago (https://www.scimagojr.com/) were hand searched between August‐October 2023 by accessing the issues list on respective journal homepages. These journals were selected based on research impact and their focus on health and performance. Knee surgery, Sports Traumatology, Arthroscopy (ranked 11/15) and Bone and Joint Journal (ranked 13/15) were excluded due to their focus on orthopaedics and surgery. Given this research focus, it was assumed that the recruitment strategies used in these journals would be less relevant to the exercise and sport performance research setting. Screening of other relevant journals including International Journal of Sport Nutrition and Exercise Metabolism, International Journal of Sport Physiology and Performance, Medicine and Science in Sport and Exercise, Journal of Sports Sciences and European Journal of Sport Science was also conducted. These journals were selected based on their health and performance focus and the lead authors' perception that a significant proportion of studies published within these journals recruit higher calibre athletes. Within the screened journals, special issues were included, whereas supplementary issues presenting conference abstracts were excluded. Letters to the editor and review papers were not analysed based on the lack of participant recruitment. A total of 2356 journal articles were screened across the 18 journals. Included journals are listed in Table S1.

Studies were included for analysis if recruited participants were aged 18 or over and considered at or above tier 3 (tier 3, highly trained/national level; tier 4, elite/international level; tier 5, world class) based on the recently proposed athlete calibre classification system (McKay et al., 2022). During the screening process, athlete calibre was first determined based on participant characteristics and descriptions. When participants were identified as meeting the tier 3 threshold, all text related to recruitment processes were extracted. Two authors conducted extractions (JR and LM). Where appropriate, author affiliations were used to assist in identifying athlete calibre and describing recruitment procedures. For example, when participants were all members of the same sporting team and author affiliation was to a related professional sporting organisation, this affiliation was used to assist in classifying athlete calibre as well as informing recruitment procedures. When no recruitment information was presented, this was noted by the researcher.

### 2.2 Analysis

The thematic analysis of the extracted text was guided by recommendations from Kiger and Varpio (Kiger et al., 2020). Extracted text was read, re‐read and then coded using an inductive approach by one author (LM). This process involved the development of codes and themes based on extracted data. A coding framework was developed during the reading and coding process, along with an audit trail of changes in coding and code refinement to maintain transparency of the analysis process. Semantic content themes were developed from codes using an interpretivist approach, with a thematic map produced to visualise connections between themes and subthemes. After themes had been developed, they were shared with a second author (AF) who appraised the alignment of coded data within the developed themes, providing recommendations for amendments to themes as appropriate. Once recommendations were addressed, the first author conducted a follow‐up review of themes to ensure coherence within each theme and distinctness between themes. Themes were then named and defined, before being shared with all authors for final confirmation. Coding and thematic analysis was conducted using qualitative data analysis software (NVivo version 14.0, QSR International PTY Ltd., Doncaster, Australia 2012). Ethical approval was not required for this study.

### 2.3 Position of the researchers

It is noted that the experience and perspectives of the researchers will influence their interpretation and analysis of extracted data. Specifically, LM has previously conducted research, including qualitative research, with athletes and used a variety of recruitment strategies with varied success. These include contacting sporting organisations and coaches to access athletes, using social media, using existing networks of the research group and recruiting athletes at competition. In addition to these approaches, AF has supervised doctoral candidates embedded as research practitioners within elite sporting teams, where recruitment into research projects occurred directly through the sporting team. AF has extensive experience conducting qualitative research. LB is a long‐term sports science researcher, having conducted projects both from within the high‐performance sporting system as well as academia, with participants ranging from Tier 3 (highly trained) to Tier 5 (world class calibre). LB has pioneered new recruitment strategies such as the research‐embedded training camp protocol (McKay et al., 2023) and is particularly focussed on the lack of female representation in sports science research (Smith et al., 2022). JR is a novice researcher and has no experience with athlete recruitment. LM, LB and AF have previously experienced challenges recruiting athletes into research. This experience provided the motivation for the current study, with the intention to inform both their own approach and the approach taken by other researchers to conduct research with athletes.

## 3 RESULTS

**FIGURE 1:**
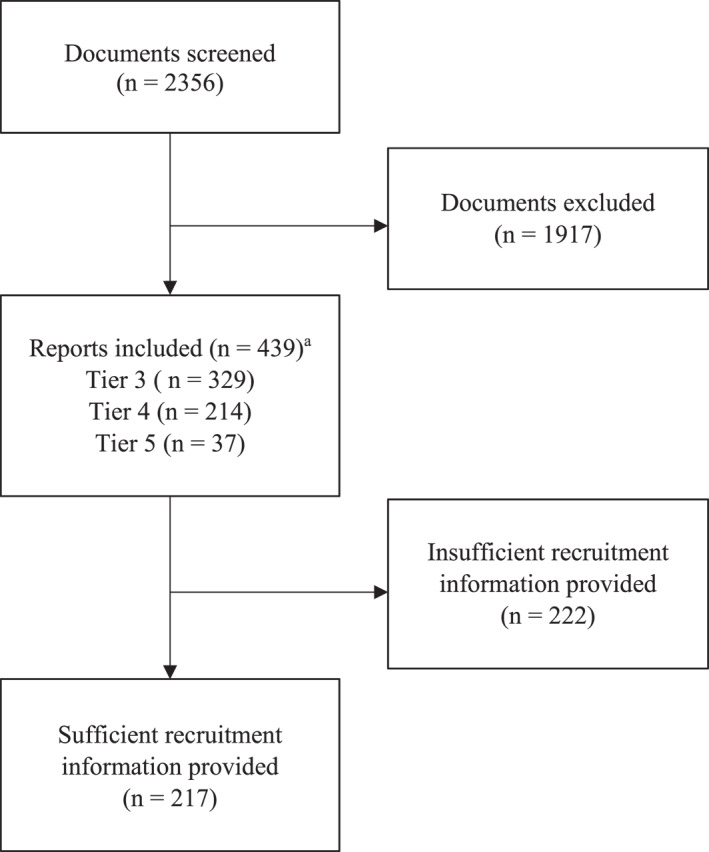
Document screening flow chart. ^a^ studies recruited athletes from multiple tiers.

A total of 439 screened studies which had recruited athletes from tier 3 or above were included in the data analysis (Figure 1) and are listed in Table S2. Of these studies, 222 provided insufficient detail to determine how athlete recruitment occurred. 114 of the 439 included studies recruited athletes from a single team or club. Following thematic analysis, one overarching theme was identified, under which five major recruitment themes were developed (Table 1). Table 2 presents indicative quotes of each theme from included studies.

**TABLE 1:** Recruitment strategy themes that emerged from the document analysis.

Overarching theme	Major themes	Linked code	Description
Purposeful, convenience sampling	Access athletes through a gatekeeper	Author affiliated with the sporting organisation	A gatekeeper acts as a conduit by engaging the researcher with athletes. The gatekeeper could be a person, persons, organisation, or database. Most often, this gatekeeper was one of the researchers affiliated with the sporting team or organisation from which athletes were recruited.
		Initial contact with the organisation	
		Person within the organisation recruited participants	
		Recruited through national sporting organisation	
		Recruited through an athlete database	
	Research environment provides convenient access to athletes	Recruited athletes from within the university	In certain research settings, there is convenient access to elite athletes. The university research setting may provide access to elite student‐athletes to recruit into research.
		Recruited through research laboratory	
		Athlete approached researchers	
		Follow up study recruiting same participants	
	Promote the study using electronic communication formats	Social media	Distributing study details including survey links online through social media. Twitter, Instagram and Facebook were major forums for sharing information.
		Survey shared online	
	Researchers utilise their professional networks to recruit athletes	Professional network of researchers	Previous research or other work conducted with sporting clubs, organisations, or institutes allows access to athletes to conduct research.
		Used established contacts with clubs	
	Recruit athletes at competition or training	Direct contact during training	Directly contacting athletes at training or competition to provide study details and recruit.

**TABLE 2:** Indicative quotes for themes extracted from documents.

Major themes	Linked code	Indicative quote
Access athletes through a gatekeeper	Author affiliated with the sporting organisation	‘Redman is also with the Performance Science Dept, Brisbane Broncos Rugby League Club, Brisbane, Australia’ Redman, K. J., Wade, L., Kelly, V. G., Connick, M. J., & Beckman, E. M. (2022). Effects of the Off‐Season on Muscular Power in Professional Rugby League. International Journal of Sports Physiology and Performance, 17(5), 733–738.
	Initial contact with the organisation	‘Between 12 and 18 months before each of the Summer and Winter Paralympic Games between 2011 and 2020, we asked the national team coaches of all relevant sports to provide a list of athletes whom they considered to be candidates to qualify’. Steffen, K., Clarsen, B., Gjelsvik, H., Haugvad, L., Koivisto‐Mørk, A., Bahr, R., & Berge, H. M. (2022). Illness and injury among Norwegian Para athletes over five consecutive Paralympic Summer and Winter Games cycles: prevailing high illness burden on the road from 2012 to 2020. British Journal of Sports Medicine, 56(4), 204–212.
		‘The first author emailed all 24 clubs that make up the WSL (*n* = 12 clubs) and Women's Championship (*n* = 12 clubs) regarding the study by using the email(s) provided on the club websites. Where possible, the email was sent directly to first team/general managers, welfare officers, sport psychologists and other support staff members asking them to act as mediators for accessing potential participants’. Carly Perry, Aiden J. Chauntry & Francesca M. Champ (2022) Elite female footballers in England: An exploration of mental ill‐health and help‐seeking intentions, Science and Medicine in Football, 6:5, 650–659.
	Person within the organisation recruit participants	‘Athlete recruitment was facilitated through the national teams' respective contact person each season’. Stenseth, O. M. R., Barli, S. F., Martin, R. K., & Engebretsen, L. (2022). Injuries in elite women's ski jumping: a Cohort study following three International Ski Federation (FIS) World Cup seasons from 2017 to 2018 to 2019–2020. British Journal of Sports Medicine, 56(1), 35–40.
		‘Eight elite female national teams (Australia, Canada, Denmark, Iceland, Netherlands, Norway, Portugal and Sweden), competing in the 25th edition of the Algarve Cup, were invited to participate in the study. The invitation was addressed to the respective team physician who distributed the questionnaires’. Oliveira, C. B., Sousa, M., Abreu, R., Ferreira, Â., Figueiredo, P., Rago, V., Teixeira, V. H., & Brito, J. (2022). Dietary supplements usage by elite female football players: An exploration of current practices. Scandinavian Journal of Medicine & Science in Sports, 32 Suppl 1, 73–80.
	Recruited through national sporting organisation	‘Questionnaires were administered through an electronic management system that is utilised for routine collection of internal training loads and wellness monitoring for Australian Olympic athletes as previously described… Athletes were recruited through their relevant National Sporting Organisation who provided organisational consent’. Halson, S. L., Appaneal, R. N., Welvaert, M., Maniar, N., & Drew, M. K. (2022). Stressed and Not Sleeping: Poor Sleep and Psychological Stress in Elite Athletes Prior to the Rio 2016 Olympic Games. International Journal of Sports Physiology and Performance, 17(2), 195–202.
	Recruited through an athlete database	‘The French national ski team database was used to identify all elite skiers who raced for the team between 2011 (database first established) and 2018’. Guy, S., Fayard, J. M., Saithna, A., Bahroun, S., Ferreira, A., Carrozzo, A., De Jesus, S., Bulle, S., Vieira, T. D., & Sonnery‐Cottet, B. (2022). Risk of Graft Rupture After Adding a Lateral Extra‐articular Procedure at the Time of ACL Reconstruction: A Retrospective Comparative Study of Elite Alpine Skiers from the French National Team. The American Journal of Sports Medicine, 50(6), 1609–1617.
Research environment provides convenient access to athletes	Recruit athletes from within the university	‘Healthy active collegiate athletes between the ages of 18 and 25 years were recruited from the University of California Davis football (*n* = 18) and rugby (*n* = 19) teams and Reserve Officer Training Corps (ROTC) elite training group (*n* = 13)’. Lis, D. M., Jordan, M., Lipuma, T., Smith, T., Schaal, K., & Baar, K. (2022). Collagen and Vitamin C Supplementation Increases Lower Limb Rate of Force Development. International Journal of Sport Nutrition and Exercise Metabolism, 32(2), 65–73.
	Recruited through research laboratory	‘The information was also disseminated through faculty's laboratory, where national best endurance athletes regularly perform various testings’. Iva Jurov, Nicola Keay & Samo Rauter (2022) Reducing energy availability in male endurance athletes: a randomised trial with a three‐step energy reduction, Journal of the International Society of Sports Nutrition, 19:1, 179–195.
	Athlete approached researchers	‘The participant could not be blinded from the real aim of the study as he personally contacted our research team to try new training strategies’. Gattoni, C., Girardi, M., O’Neill, B. V., & Maria Marcora, S. (2022). Sleep Deprivation Training to Reduce the Negative Effects of Sleep Loss on Endurance Performance: A Single Case Study. International Journal of Sports Physiology and Performance, 17(3), 499–503.
	Follow up study recruiting same participants	‘The climbing group was contacted by telephone after participation in the baseline study 10 years earlier over a time period from Apr to August 2019’. Torsten Pastor, Andreas Schweizer, Lisa Reissner, Tatjana Pastor, Jörg Spörri & Stefan Fröhlich (2022) Long‐term evolution of cartilage abnormalities and osteophytes in the fingers of elite sport climbers: A cross‐sectional 10‐year follow‐up study, European Journal of Sport Science, 22:9, 1452–1458.
Promote the study using electronic communication formats	Social media	‘The survey was shared via email and personal/group messaging applications (e.g., WhatsApp, Signal and Telegram) and promoted on social media (e.g., Facebook, Twitter and Instagram) through the professional networks of the research team (e.g., clubs, federations and institutions)’. Washif, J. A., Farooq, A., Krug, I., et al. (2022). Training During the COVID‐19 Lockdown: Knowledge, Beliefs and Practices of 12,526 Athletes from 142 Countries and Six Continents. Sports Medicine (Auckland, N.Z.), 52(4), 933–948.
		‘The survey was launched online through social media (e.g., Facebook, Twitter, WhatsApp and e‐mail), opening 8 July and closing 30 September, 2020’. Romdhani, M., Rae, D. E., Nédélec, M., et al. (2022). COVID‐19 Lockdowns: A Worldwide Survey of Circadian Rhythms and Sleep Quality in 3911 Athletes from 49 Countries, with Data‐Driven Recommendations. Sports Medicine (Auckland, N.Z.), 52(6), 1433–1448.
	Survey shared online	‘Team managers were approached for the dissemination of the A‐NSKQ questionnaire and it was also shared online’. Michèle Renard, Ana Anton‐Solanas, David T. Kelly & Ciarán Ó Catháin (2022) Evaluation of nutrition knowledge in elite and sub‐elite Gaelic football players, Science and Medicine in Football, 6:1, 82–88.
Researchers utilise their professional networks to recruit athletes	Professional network of researchers	‘Participants were recruited and selected through convenience sampling from the research team's network of contacts based on previous links with the rugby‐league clubs’. Matthew Andrew, Ryan W. O’Brien, Paul R. Ford & Joe Causer (2022) Developmental activities of professional male British rugby‐league players versus controls, Science and Medicine in Football, 6:3, 381–388.
		‘In light of this experience, the participant recruitment process began by utilising the research teams' network of contacts to approach potential participants who met the criteria outlined above’. Gareth Prendergast & Luke Gibson (2022) A qualitative exploration of the use of player loans to supplement the talent development process of professional footballers in the under 23 age group of English football academies, Journal of Sports Sciences, 40:4, 422–430.
	Used established contacts with clubs	‘Senior players were recruited via established contacts at clubs located in Queensland, New South Wales and Victoria… All players within the training squad were invited to participate in the study’. Collings, T. J., Diamond, L. E., Barrett, R. S., Timmins, R. G., Hickey, J. T., DU Moulin, W. S., Williams, M. D., Beerworth, K. A., & Bourne, M. N. (2022). Strength and Biomechanical Risk factors for Noncontact ACL Injury in Elite female Footballers: A Prospective Study. Medicine and Science in Sports and Exercise, 54(8), 1242–1251.
Recruit athletes at competition or training	Direct contact during training	‘Judokas were invited to participate in our study… through a direct request at the training camps during the time of the survey (international training camp in Mittersill, Austria and training camp in Tokyo, Japan) and the Grand Prix Tel Aviv 2020 competition’. Maruša Štangar, Anja Štangar, Volha Shtyrba, Blaž Cigić & Evgen Benedik (2022) Rapid weight loss among elite‐level judo athletes: Methods and nutrition in relation to competition performance, Journal of the International Society of Sports Nutrition, 19:1, 380–396.
		‘Furthermore, the playing group was informed about the purpose of the research through a formal meeting’. Rhys Tribolet, William B. Sheehan, Andrew R. Novak, Mark L. Watsford & Job Fransen (2022) factors associated with cooperative network connectedness in a professional Australian football small‐sided game, Science and Medicine in Football, 6:4, 511–518.

Purposeful, convenience sampling was identified as the overarching recruitment strategy from the included studies and this theme links with each of the five major recruitment themes. We defined purposeful sampling as relating to the recruitment of targeted athletes based on their sport, team, or calibre. We defined convenience sampling as relating to the recruitment of athletes based on their ease of access and their location relative to the researchers.

The most prominent major theme was that athletes were accessed through a gatekeeper. This theme describes how engaging athletes was coordinated through some gatekeeper, be that a single person, persons, or an organisation. The most prominent subtheme within this constructed theme related to an author of the study being affiliated with the sporting team or organisation from which the athletes were recruited. An extension of this was the small number of studies where one of the researchers was an active or formerly active athlete within the sport. Other studies within this gatekeeper theme recruited athletes through a person or contact within the sporting organisation, such as the coach, manager, physiotherapist, or other medical personnel. Other studies simply note a ‘representative’ or ‘club contact person’ was used to recruit athletes. Recruitment through the National Sporting Organisation or Institute was also a common thread within this theme. Initial contact was typically made between researcher and organisation, followed by either the organisation contacting athletes with study information for recruitment purposes or the organisation providing contact details of athletes to researchers. Direct contact was then made, typically via email, although some face‐to‐face contact was reported. The use of an athlete database was also reported in several studies under this gatekeeper theme, including US college athlete registries, surgical databases and publicly accessible athlete ranking databases.

The second major theme was that some research environments provide convenient access to athletes. This theme describes the recruitment of athletes who typically train or perform within close proximity of the research laboratory. Most studies within this theme were conducted in the university setting with elite student‐athletes as participants. A very small number of other codes were linked to this theme, including athletes approaching the researcher to undergo investigations and conducting a follow up study of previously investigated athletes.

The third major strategy used by researchers was to promote the study using electronic communication formats. In these studies, researchers utilised primarily social media to disseminate information about a study in attempts to recruit athletes. Only a small number of studies used this strategy (*n* = 22) and most of these studies asked participants to complete an online survey on one or multiple occasions. In addition, several of these studies recruited both recreational and highly trained athletes. The use of online promotion of the study also involved some direct contact with athletes, whereby researchers sent direct messages to athletes via social media with study information.

The fourth major theme involved the use of professional networks to recruit athletes. Connections related to the researchers' previous research or other work conducted with sporting clubs, organisations, or institutions, provided an avenue to engage athletes into the research environment as participants. For example, ‘Participants were recruited and selected through convenience sampling from the research team's network of contacts based on previous links with the rugby league clubs’ (Andrew et al., 2022). Networks were also highlighted when circulating study details via social media or email.

The final theme was to recruit athletes at competition or training. A small number of studies engaged athletes directly at either a training venue, including training camp, or at competition, to promote the study and recruit participants.

## 4 DISCUSSION

The aim of this document analysis was to identify strategies used in sport science research to engage and recruit highly trained, elite and world class athletes as participants. Through the analysis of 439 journal articles, five main recruitment strategies were identified, with an additional overarching theme describing the recruitment approach as purposeful and convenient. Accessing athletes through a gatekeeper was the predominant strategy used, with other strategies relating to convenient access to elite athletes at research facilities, the use of social media to recruit and distribute study information, relying on previously formed networks and attending training and competition to recruit.

The most prominent recruitment strategy identified from the analysed studies was the use of a gatekeeper to engage and recruit athletes. Within this theme, having an author affiliated with the sporting team or organisation was the most widely used approach. The document analysis was unable to determine the origin of the affiliation: whether the researcher moved into the sporting organisation or whether the research group invited personnel from within the sporting organisation to collaborate on the project. Either approach would appear to be a viable and successful means to conduct research with elite level athletes. Embedding a research member within the sporting organisation can be a successful approach to recruiting athletes into research (Coutts, 2016). This method also facilitates the co‐design of research with end‐users, ensuring that research questions are of authentic interest to the athletes and coaches to promote their engagement, while safeguarding the logistics around the implementation of protocols (McKay et al., 2023). Embedded researchers are able to identify system and environmental constraints that may limit the application of findings or methods from traditional research (Coutts, 2016). This may result in designing protocols that are better able to measure metrics of interest, via direct characteristics of superior reliability and ecological validity, as well as the genuine engagement of participants. Indeed, some studies have incorporated pre‐experimental control of diet and exercise within actual sporting competitions to monitor changes in performance (Burke et al., 2017), representing the merging of ideal research features with intrinsic motivation and real‐world conditions. Truly embedded research models have been shown to provide additional value to the incumbent participants and sports; for example, providing funding that supports otherwise unaffordable training camps, or allowing access to novel sports science or coaching expertise, testing, or resources (McKay et al., 2023). Ultimately, conducting research with individuals within the sporting organisation, whether an embedded researcher or otherwise, appears to improve recruitment capacity while also increasing the relevance and translation of sport science research with the elite athlete (Coutts, 2017).

Beyond the sporting organisation‐affiliated author, the gatekeeper took on the form of staff within an organisation or the organisation itself. Staff identified in the document analysis as potential gatekeepers were coaches, managers and medical personnel. Engaging these key stakeholders may provide more opportunity to involve athletes in the research environment. When coaches and staff see value in the research project, they may be more likely to endorse the project. These stakeholders also provide an opportunity for research co‐design, leading to greater outcomes for athletes, staff and researchers (Coutts, 2017). The organisation was identified as a gatekeeper in a small number of analysed studies. National Sporting Organisations, governing bodies and Institutes of Sport were most commonly utilised as points of contact to then gain access to athletes. Although this method was an important strategy identified from this recruitment theme, the small number of studies reporting this approach may reflect its feasibility, particularly given the privacy and consent requirements around providing athlete contact details to researchers.

Researchers utilised their local environment to recruit athletes as participants. This strategy primarily involved the recruitment of elite student‐athletes in the university setting. Making use of resources available within the research environment seems an intuitive decision to assist recruitment. When elite athletes grace the hallways and practice fields outside the research laboratory, it makes sense to engage these athletes as potential participants in research. Many of the studies that employed this recruitment strategy had authors affiliated with the university sporting team or department, again suggesting some form of gatekeeper may have been involved in study design and recruitment. Beyond these studies, many other studies screened in the document analysis recruited university athletes but did not supply sufficient information to determine how recruitment took place. Clearly then, student‐athletes can be an accessible cohort to involve in sport science research. The calibre of athletes may vary, but certainly the NCAA system in the United States has a large pool of highly trained and elite athletes that could be engaged with as research participants (McKay et al., 2022). Including team staff in the research process also opens up co‐design opportunities that can increase research translation (Grindell et al., 2022).

A noteworthy finding was the use of online and social media promotion of studies to recruit participants. Social media has been recognised as an emerging resource for participant recruitment in health research, with suggestions social media may be an effective platform for accessing hard‐to‐reach populations (Gelinas et al., 2017). However, social media also has its limitations as a recruitment strategy, including samples unrepresentative of the target population (Arigo et al., 2018). A scoping review of social media recruitment for medical research showed 12 of 30 studies found social media to be the most effective recruitment method, eight of which were observational studies (Topolovec‐Vranic et al., 2016). Findings from the current document analysis seem to support this, with most studies recruiting through social media using online surveys to collect data. It is also important to note that many of the screened studies in the current document analysis were conducted in 2020 during the COVID‐19 pandemic when face‐to‐face research was severely limited. As such, study designs and recruitment methods likely shifted in response to the restrictions in place at the time.

Researchers utilised their professional network to assist in participant recruitment in a small number of studies. Networks appear to have been developed through previous research or work with sporting teams and organisations. In these scenarios, researchers may potentially bypass the requirement for a gatekeeper to gain access to athletes, having previously built rapport with staff and athletes themselves. Many of the studies that employed this strategy also utilised social media to recruit athletes. This suggests researchers with a wider following and previous connections with athletes and teams can lean on these networks to recruit for future research. Although a viable strategy, it is likely unavailable to research groups yet to develop a track record of research in the elite sporting environment. As such, its broader applicability as a recruitment strategy for researchers may be limited. A potential solution could be to collaborate with other researchers who already have developed networks; these researchers themselves acting as gatekeepers.

The current study has several strengths. A rigorous, systematic approach was taken in the document analysis, with a large sample of studies screened to obtain relevant data. Furthermore, the thematic analysis was performed in accordance with recommended procedures, improving transparency and trustworthiness of the findings (Cleland et al., 2023; Kiger et al., 2020). There are limitations that should be considered when interpreting findings. The selection of journals for document screening was based on impact factor. Many of the included journals have a sports medicine focus, where particular recruitment strategies may be more effective than in sports performance research. The studies included in the document analysis were published subsequent to a unique time in science. Specifically, the COVID‐19 pandemic had a substantial impact on research opportunities and this likely influenced the design of many studies included in this document analysis and as an extension, the recruitment strategies used. Although several meaningful themes were constructed from the document analysis, over half of the included studies provided insufficient detail to determine how recruitment took place. Therefore, there may be other recruitment strategies not described in the current analysis that could be considered for future sport science research. This limitation reinforces the importance of transparent reporting in sport science research (Halperin et al., 2018).

## 5 CONCLUSION

Purposeful and convenient recruitment appear to be the primary broad approaches taken to recruit well trained, elite and world class athletes as research participants. Strategies identified in the document analysis included the use of a gatekeeper, recruiting athletes that are commonly within close proximity of the research setting, promoting the study on social media, utilising researcher networks and recruiting athletes at training or competition. It is suggested that researchers work collaboratively with team or organisation personnel to engage athletes and consider co‐designed approaches that address issues most relevant to athletes and staff.

## CONFLICT OF INTEREST STATEMENT

The authors report there are no competing interests to declare.

## PARTICIPANT CONSENT STATEMENT

No participants were recruited for this study.

## PERMISSION TO REPRODUCE

No material was reproduced in the production of this manuscript.

## Supporting information

Table S1

Table S2

## Data Availability

The data that support the findings of this study are available from the corresponding author upon reasonable request.
