# Supplementary material for: Engaging athletes as research participants. A document analysis of published sport science literature
Source: Eur J Sport Sci. 2024 Sep 17;24(10):1442–51. doi: 10.1002/ejsc.12198 (PMC11451557; doi:10.1002/ejsc.12198)
Supplement: Supplementary file 1 — Table S1 [file EJSC-24-1442-s002.docx]

Supplementary Table 1. Journals screened and characteristics of studies included in the document analysis.

| Journal | Journal aim | Screened documents (n) | Included documents (n) | Athlete calibre of included documents (n)^a^ | | |
| --- | --- | --- | --- | --- | --- | --- |
|  |  |  |  | Tier 3 | Tier 4 | Tier 5 |
| British Journal of Sports Medicine | Provides original research, reviews and debate relating to clinically relevant aspects of sport and exercise medicine, including physiotherapy, physical therapy, and rehabilitation | 227 | 13 | 8 | 5 | 2 |
| Sports Medicine | Focuses on definitive and comprehensive review articles that interpret and evaluate the current literature to provide the rationale for and application of research findings in the sports medicine and exercise field. Also publishes high-quality original research articles. | 167 | 8 | 4 | 5 | 1 |
| Exercise Immunology Reviews | Committed to developing and enriching knowledge in all aspects of immunology that relate to sport, exercise, and regular physical activity. | 7 | 0 | 0 | 0 | 0 |
| Qualitative Research in Sport, Exercise, and Health | Providing a forum for qualitative researchers within all the social scientific areas of sport, exercise, and health the journal offers researchers, practitioners, and students access to cutting edge empirical inquiry, scholarly dialogues, and the latest developments in qualitative methodologies and methods. | 69 | 6 | 3 | 4 | 1 |
| International Review of Sport and Exercise Psychology | Publishes critical reviews of research literature in sport and exercise psychology. Typically, these reviews evaluate relevant conceptual and methodological issues in the field and provide a critique of the strengths and weaknesses of empirical studies that address common themes or hypotheses. | 12 | 0 | 0 | 0 | 0 |
| Biology of Sport | The journal publishes articles concerning basic and applied sciences in sport: sports and exercise physiology, sports genetics, training and testing, sports performance and analysis, as well as in other biological aspects related to sport. | 104 | 39 | 29 | 15 | 3 |
| Sport Management Review | A multidisciplinary journal concerned with the management, marketing, and governance of sport at all levels and in all its manifestations -- whether as an entertainment, a recreation, or an occupation. | 38 | 2 | 2 | 1 | 0 |
| Journal of the International Society of Sport Nutrition | Focuses on the acute and chronic effects of sports nutrition and supplementation strategies on body composition, physical performance, and metabolism | 37 | 9 | 7 | 6 | 0 |
| Exercise and Sport Sciences Reviews | The mission of this American College of Sports Medicine publication is to provide premier, peer-reviewed quarterly reviews of the most contemporary scientific, medical, and research-based topics emerging in the field of sports medicine and exercise science. | 25 | 0 | 0 | 0 | 0 |
| American Journal of Sports Medicine | Contains original articles addressed to orthopaedic surgeons specializing in sports medicine, and to team physicians, athletic trainers, and physical therapists focusing on the causes and effects of injury or disease. | 391 | 19 | 13 | 14 | 2 |
| Science and Medicine in Football | The mission of the publication is to advance the theoretical knowledge, methodological approaches and the professional practice associated with the sport of football. The journal aims to publish meaningful articles on various aspects of sports medicine and science related to all codes of football. | 74 | 39 | 36 | 9 | 1 |
| Scandinavian Journal of Medicine and Science in Sport | It aims to publish high quality and impactful articles in the fields of orthopaedics, rehabilitation and sports medicine, exercise physiology and biochemistry, biomechanics and motor control, health and disease relating to sport, exercise and physical activity, as well as on the social and behavioural aspects of sport and exercise. | 157 | 24 | 17 | 11 | 2 |
| International Journal of Sport and Exercise Psychology | publishes empirical and theoretical contributions in the science of physical activity, human movement, exercise, and sport. The primary purposes of IJSEP are to promote understanding of sport psychology research and practice around the world, enhance theoretical and practical knowledge in these fields and promote high-quality scientific and applied work in sport and exercise psychology. | 98 | 24 | 21 | 13 | 2 |
| European Journal of Sport Science | To promote the highest standards of scientific study and scholarship in respect of the following fields: (a) Applied Sport Sciences; (b) Biomechanics and Motor Control; c) Physiology and Nutrition; (d) Psychology, Social Sciences and Humanities and (e) Sports and Exercise Medicine and Health. | 196 | 57 | 40 | 26 | 3 |
| Journal of Sports Sciences | It publishes research on various aspects of the sports and exercise sciences, including anatomy, biochemistry, biomechanics, performance analysis, physiology, psychology, sports medicine and health, as well as coaching and talent identification, kinanthropometry and other interdisciplinary perspectives. | 274 | 57 | 49 | 28 | 4 |
| Medicine and Science in Sport and Exercise | Features original investigations, clinical studies, and comprehensive reviews on current topics in sports medicine and exercise science. | 221 | 27 | 23 | 15 | 0 |
| International Journal of Sport Physiology and Performance | Focuses on sport physiology and performance and is dedicated to advancing the knowledge of sport and exercise physiologists, sport-performance researchers, and other sport scientists. The journal publishes authoritative peer-reviewed research in sport physiology and related disciplines, with an emphasis on work having direct practical applications in enhancing sport performance. | 212 | 109 | 73 | 59 | 14 |
| International Journal of Sport Nutrition and Exercise Metabolism | Publishes original scientific investigations and scholarly reviews offering new insights into sport nutrition and exercise metabolism, as well as articles focusing on the application of the principles of biochemistry, physiology, and nutrition to sport and exercise. | 47 | 6 | 4 | 3 | 0 |

Journal aim has been extracted from the respective journal websites. ^a^, value refers to the number of studies that had participants of the stated tier; studies recruited athletes from multiple tiers.
