# Supplementary material for: Engaging athletes as research participants. A document analysis of published sport science literature
Source: Eur J Sport Sci. 2024 Sep 17;24(10):1442–51. doi: 10.1002/ejsc.12198 (PMC11451557; doi:10.1002/ejsc.12198)
Supplement: Supplementary file 2 — Table S2 [file EJSC-24-1442-s001.docx]

**Supplementary Table 2.** Documents included in analysis

| 1. Tabben, M., Eirale, C., Singh, G., Al-Kuwari, A., Ekstrand, J., Chalabi, H., Bahr, R., & Chamari, K. (2022). Injury and illness epidemiology in professional Asian football: lower general incidence and burden but higher ACL and hamstring injury burden compared with Europe. British journal of sports medicine, 56(1), 18–23. https://doi.org/10.1136/bjsports-2020-102945 |
| --- |
| 1. Stenseth, O. M. R., Barli, S. F., Martin, R. K., & Engebretsen, L. (2022). Injuries in elite women's ski jumping: a cohort study following three International Ski Federation (FIS) World Cup seasons from 2017-2018 to 2019-2020. British journal of sports medicine, 56(1), 35–40. https://doi.org/10.1136/bjsports-2021-104198 |
| 1. Harmon, K. G., Whelan, B. M., Aukerman, D. F., Bohr, A. D., Nerrie, J. M., Elkinton, H. A., Holliday, M., Poddar, S. K., Chrisman, S. P. D., & McQueen, M. B. (2022). Diagnostic accuracy and reliability of sideline concussion evaluation: a prospective, case-controlled study in college athletes comparing newer tools and established tests. British journal of sports medicine, 56(3), 144–150. https://doi.org/10.1136/bjsports-2020-103840 |
| 1. Tso, J. V., Turner, C. G., Liu, C., Galante, A., Gilson, C. R., Clark, C., Taylor, H. A., Quyyumi, A. A., Baggish, A. L., & Kim, J. H. (2022). Association between race and maladaptive concentric left ventricular hypertrophy in American-style football athletes. British journal of sports medicine, 56(3), 151–157. https://doi.org/10.1136/bjsports-2021-104333 |
| 1. Steffen, K., Clarsen, B., Gjelsvik, H., Haugvad, L., Koivisto-Mørk, A., Bahr, R., & Berge, H. M. (2022). Illness and injury among Norwegian Para athletes over five consecutive Paralympic Summer and Winter Games cycles: prevailing high illness burden on the road from 2012 to 2020. British journal of sports medicine, 56(4), 204–212. https://doi.org/10.1136/bjsports-2021-104489 |
| 1. Pollock, N., Kelly, S., Lee, J., Stone, B., Giakoumis, M., Polglass, G., Brown, J., & MacDonald, B. (2022). A 4-year study of hamstring injury outcomes in elite track and field using the British Athletics rehabilitation approach. British journal of sports medicine, 56(5), 257–263. https://doi.org/10.1136/bjsports-2020-103791 |
| 1. Silva, A. M., Nunes, C. L., Jesus, F., Francisco, R., Matias, C. N., Cardoso, M., Santos, I., Carraça, E. V., Finlayson, G., Silva, M. N., Dickinson, S., Allison, D., Minderico, C. S., Martins, P., & Sardinha, L. B. (2022). Effectiveness of a lifestyle weight-loss intervention targeting inactive former elite athletes: the Champ4Life randomised controlled trial. British journal of sports medicine, 56(7), 394–401. https://doi.org/10.1136/bjsports-2021-104212 |
| 1. Racinais, S., Havenith, G., Aylwin, P., Ihsan, M., Taylor, L., Adami, P. E., Adamuz, M. C., Alhammoud, M., Alonso, J. M., Bouscaren, N., Buitrago, S., Cardinale, M., van Dyk, N., Esh, C. J., Gomez-Ezeiza, J., Garrandes, F., Holtzhausen, L., Labidi, M., Lange, G., Lloyd, A., … Bermon, S. (2022). Association between thermal responses, medical events, performance, heat acclimation and health status in male and female elite athletes during the 2019 Doha World Athletics Championships. British journal of sports medicine, 56(8), 439–445. https://doi.org/10.1136/bjsports-2021-104569 |
| 1. Davenport, M. H., Nesdoly, A., Ray, L., Thornton, J. S., Khurana, R., & McHugh, T. F. (2022). Pushing for change: a qualitative study of the experiences of elite athletes during pregnancy. British journal of sports medicine, 56(8), 452–457. https://doi.org/10.1136/bjsports-2021-104755 |
| 1. Ruffault, A., Sorg, M., Martin, S., Hanon, C., Jacquet, L., Verhagen, E., & Edouard, P. (2022). Determinants of the adoption of injury risk reduction programmes in athletics (track and field): an online survey of 7715 French athletes. British journal of sports medicine, 56(9), 499–505. https://doi.org/10.1136/bjsports-2021-104593 |
| 1. Farley, T., Barry, E., Sylvester, R., Medici, A., & Wilson, M. G. (2022). Poor isometric neck extension strength as a risk factor for concussion in male professional Rugby Union players. British journal of sports medicine, 56(11), 616–621. https://doi.org/10.1136/bjsports-2021-104414 |
| 1. Wezenbeek, E., Denolf, S., Willems, T. M., Pieters, D., Bourgois, J. G., Philippaerts, R. M., De Winne, B., Wieme, M., Van Hecke, R., Markey, L., Schuermans, J., Witvrouw, E., & Verstockt, S. (2022). Association between SARS-COV-2 infection and muscle strain injury occurrence in elite male football players: a prospective study of 29 weeks including three teams from the Belgian professional football league. British journal of sports medicine, bjsports-2021-104595. Advance online publication. https://doi.org/10.1136/bjsports-2021-104595 |
| 1. Bruce, J. M., Meeuwisse, W., Hutchison, M. G., Comper, P., & Echemendia, R. J. (2022). Determining Sport Concussion Assessment Tool fifth Edition (SCAT5) reliable change in male professional hockey players. British journal of sports medicine, bjsports-2021-104851. Advance online publication. https://doi.org/10.1136/bjsports-2021-104851 |
| 1. Lempke, L. B., Lynall, R. C., Anderson, M. N., McCrea, M. A., McAllister, T. W., Broglio, S. P., Schmidt, J. D., & CARE Consortium Investigators (2022). Optimizing Order of Administration for Concussion Baseline Assessment Among NCAA Student-Athletes and Military Cadets. Sports medicine (Auckland, N.Z.), 52(1), 165–176. https://doi.org/10.1007/s40279-021-01493-y |
| 1. Lievens, E., Van Vossel, K., Van de Casteele, F., Wezenbeek, E., Deprez, D., Matthys, S., De Winne, B., McNally, S., De Graaf, W., Murdoch, J. B., Bourgois, J. G., Witvrouw, E., & Derave, W. (2022). Muscle Fibre Typology as a Novel Risk Factor for Hamstring Strain Injuries in Professional Football (Soccer): A Prospective Cohort Study. Sports medicine (Auckland, N.Z.), 52(1), 177–185. https://doi.org/10.1007/s40279-021-01538-2 |
| 1. Halson, S. L., Johnston, R. D., Appaneal, R. N., Rogers, M. A., Toohey, L. A., Drew, M. K., Sargent, C., & Roach, G. D. (2022). Sleep Quality in Elite Athletes: Normative Values, Reliability and Understanding Contributors to Poor Sleep. Sports medicine (Auckland, N.Z.), 52(2), 417–426. https://doi.org/10.1007/s40279-021-01555-1 |
| 1. Washif, J. A., Farooq, A., Krug, I., Pyne, D. B., Verhagen, E., Taylor, L., Wong, D. P., Mujika, I., Cortis, C., Haddad, M., Ahmadian, O., Al Jufaili, M., Al-Horani, R. A., Al-Mohannadi, A. S., Aloui, A., Ammar, A., Arifi, F., Aziz, A. R., Batuev, M., Beaven, C. M., … Chamari, K. (2022). Training During the COVID-19 Lockdown: Knowledge, Beliefs, and Practices of 12,526 Athletes from 142 Countries and Six Continents. Sports medicine (Auckland, N.Z.), 52(4), 933–948. https://doi.org/10.1007/s40279-021-01573-z |
| 1. Romdhani, M., Rae, D. E., Nédélec, M., Ammar, A., Chtourou, H., Al Horani, R., Ben Saad, H., Bragazzi, N., Dönmez, G., Driss, T., Fullagar, H. H. K., Farooq, A., Garbarino, S., Hammouda, O., Hassanmirzaei, B., Khalladi, K., Khemila, S., Mataruna-Dos-Santos, L. J., Moussa-Chamari, I., Mujika, I., … Chamari, K. (2022). COVID-19 Lockdowns: A Worldwide Survey of Circadian Rhythms and Sleep Quality in 3911 Athletes from 49 Countries, with Data-Driven Recommendations. Sports medicine (Auckland, N.Z.), 52(6), 1433–1448. https://doi.org/10.1007/s40279-021-01601-y |
| 1. Eagle, S. R., Asken, B., Trbovich, A., Houck, Z. M., Bauer, R. M., Clugston, J. R., Broglio, S. P., McAllister, T. W., McCrea, M. A., Pasquina, P., Collins, M. W., Kontos, A. P., & CARE Consortium Investigators (2022). Estimated Duration of Continued Sport Participation Following Concussions and Its Association with Recovery Outcomes in Collegiate Athletes: Findings from the NCAA/DoD CARE Consortium. Sports medicine (Auckland, N.Z.), 52(8), 1991–2001. https://doi.org/10.1007/s40279-022-01668-1 |
| 1. Valle, X., Mechó, S., Alentorn-Geli, E., Järvinen, T. A. H., Lempainen, L., Pruna, R., Monllau, J. C., Rodas, G., Isern-Kebschull, J., Ghrairi, M., Yanguas, X., Balius, R., & la Torre, A. M. (2022). Return to Play Prediction Accuracy of the MLG-R Classification System for Hamstring Injuries in Football Players: A Machine Learning Approach. Sports medicine (Auckland, N.Z.), 52(9), 2271–2282. https://doi.org/10.1007/s40279-022-01672-5 |
| 1. Moody, J. N., Hayes, J. P., Buckley, T. A., Schmidt, J. D., Broglio, S. P., McAllister, T. W., McCrea, M., Pasquina, P. F., Caccese, J. B., & CARE Consortium Investigators (2022). Age of First Concussion and Cognitive, Psychological, and Physical Outcomes in NCAA Collegiate Student Athletes. Sports medicine (Auckland, N.Z.), 52(11), 2759–2773. https://doi.org/10.1007/s40279-022-01719-7 |
| 1. Kasper Bluhm & Susanne Ravn (2022) ‘It has to hurt’: A phenomenological analysis of elite runners´ experiences in handling non-injuring running-related pain, Qualitative Research in Sport, Exercise and Health, 14:2, 216-231 |
| 1. Donka Darpatova-Hruzewicz (2022) Reflexive confessions of a female sport psychologist: from REBT to existential counselling with a transnational footballer, Qualitative Research in Sport, Exercise and Health, 14:2, 306-325 |
| 1. Monica Nelson & Shannon Jette (2022) Down with the Thickness?: Male Olympic Weightlifters’ Negotiations of Weight Class, Strength, & Body Composition, Qualitative Research in Sport, Exercise and Health, 14:4, 580-595, |
| 1. Holly Bradshaw, Karen Howells & Mathijs Lucassen (2022) Abandoned to manage the post-Olympic blues: Olympians reflect on their experiences and the need for a change, Qualitative Research in Sport, Exercise and Health, 14:5, 706-723, |
| 1. Jannika M. John & Ansgar Thiel (2022) All roads lead to Rome? Talent narratives of elite athletes, musicians, and mathematicians, Qualitative Research in Sport, Exercise and Health, 14:7, 1174-1195, |
| 1. Carly Harrison, Mandy Ruddock-Hudson, Sue Mayes, Paul O’Halloran, Katia Ferrar, Scott Ruddock & Jill Cook (2022) An exploration of the perceptions and experiences of professional ballet dancers using a wellness monitoring application, Qualitative Research in Sport, Exercise and Health, 14:7, 1196-1212, |
| 1. Souissi, W., Hammouda, O., Ammar, A., Ayachi, M., Bardiaa, Y., & Daoud, O. et al. (2022). Higher evening metabolic responses contribute to diurnal variation of self-paced cycling performance. Biology of Sport, 39(1), 3-9. https://doi.org/10.5114/biolsport.2021.102930 |
| 1. Alexander, J., Keegan, J., Reedy, A., & Rhodes, D. (2022). Effects of contemporary cryo-compression on post-training performance in elite academy footballers. Biology of Sport, 39(1), 11-17. |
| 1. Alexander, J., Carling, C., & Rhodes, D. (2022). Utilisation of performance markers to establish the effectiveness of cold-water immersion as a recovery modality in elite football. Biology of Sport, 39(1), 19-29. https://doi.org/10.5114/biolsport.2021.103570 |
| 1. J. Malone, J., Hodges, D., Roberts, C., K. Sinclair, J., M. Page, R., & Allan, R. (2022). Effect of alterations in whole-body cryotherapy (WBC) exposure on post-match recovery markers in elite Premier League soccer players. Biology of Sport, 39(1), 31-36. https://doi.org/10.5114/biolsport.2021.102931 |
| 1. Filter-Ruger, A., Gantois, P., S. Henrique, R., Olivares-Jabalera, J., Robles-Rodríguez, J., & Santalla, A. et al. (2022). How does curve sprint evolve across different age-categories  in soccer players?. Biology of Sport, 39(1), 53-58. https://doi.org/10.5114/biolsport.2022.102867 |
| 1. Moura Zagatto, A., Medeiros Dutra, Y., Claus, G., de Sousa Malta, E., Bonetti. de Poli, R., & Pinheiro Brisola, G. et al. (2022). Drop jumps improve repeated sprint ability performances in professional basketball players. Biology of Sport, 39(1), 59-66. https://doi.org/10.5114/biolsport.2021.101128 |
| 1. Casamichana, D., Martín-García, A., Gómez Díaz, A., S Bradley, P., & Castellano, J. (2022). Accumulative weekly load in a professional football team: with special reference to match playing time and game position. Biology of Sport, 39(1), 115-124. https://doi.org/10.5114/biolsport.2021.102924 |
| 1. M. Oliva Lozano, J., Rago, V., Fortes, V., & M. Muyor, J. (2022). Impact of match-related contextual variables on weekly training load in a professional soccer team: a full season study. Biology of Sport, 39(1), 125-134. https://doi.org/10.5114/biolsport.2021.102927 |
| 1. Dello Iacono, A., Unnithan, V., Shushan, T., King, M., & Beato, M. (2022). Training load responses to football game profile-based training (GPBT) formats: effects of locomotive demands manipulation. Biology of Sport, 39(1), 145-155. https://doi.org/10.5114/biolsport.2021.102919 |
| 1. García, F., Schelling, X., Castellano, J., Martín-García, A., Pla, F., & Vázquez-Guerrero, J. (2022). Comparison of the most demanding scenarios during different in-season training sessions and official matches in professional basketball players. Biology of Sport, 39(2), 237-244. https://doi.org/10.5114/biolsport.2022.104064 |
| 1. Gam, S., K. Klitgaard, K., B. Funch, A., E. Sloth, M., W. Holt, J., & L. Molbech, J. et al. (2022). Optimal and freely chosen paddling rate during moderate kayak ergometry. Biology of Sport, 39(2), 289-293. https://doi.org/10.5114/biolsport.2022.104915 |
| 1. Djaoui, L., Owen, A., Newton, M., Theodoros Nikolaidis, P., Dellal, A., & Chamari, K. (2022). Effects of congested match periods on acceleration and deceleration profiles in professional soccer. Biology of Sport, 39(2), 307-317. https://doi.org/10.5114/biolsport.2022.103725 |
| 1. Selmi, O., Ouergui, I., E Levitt, D., Marzouki, H., Knechtle, B., & Nikolaidis, P. et al. (2022). Training, psychometric status, biological markers and neuromuscular fatigue in soccer. Biology of Sport, 39(2), 319-327. https://doi.org/10.5114/biolsport.2022.104065 |
| 1. Spyrou, K., T. Freitas, T., Marín-Cascales, E., Herrero-Carrasco, R., & E. Alcaraz, P. (2022). External match load and the influence of contextual factors in elite futsal. Biology of Sport, 39(2), 349-354. https://doi.org/10.5114/biolsport.2022.105332 |
| 1. Petr, M., Thiel, D., Kateřina, K., Brož, P., Malý, T., & Zahálka, F. et al. (2022). Speed and power-related gene polymorphisms associated with playing position in elite soccer players. Biology of Sport, 39(2), 355-366. https://doi.org/10.5114/biolsport.2022.105333 |
| 1. M. Oliva-Lozano, J., Gómez-Carmona, C., Fortes, V., & Pino-Ortega, J. (2022). Effect of training day, match, and length of the microcycle on workload periodization in professional soccer players: a full-season study. Biology of Sport, 39(2), 397-406. https://doi.org/10.5114/biolsport.2022.106148 |
| 1. Kikuchi, N., Tajima, T., Tamura, Y., Yamanaka, Y., Menuki, K., & Okamoto, T. et al. (2022). The ALDH2 rs671 polymorphism is associated with athletic status  and muscle strength in a Japanese population. Biology of Sport, 39(2), 429-434. https://doi.org/10.5114/biolsport.2022.106151 |
| 1. Thoseby, B., D. Govus, A., C. Clarke, A., J. Middleton, K., & J. Dascombe, B. (2022). Between-match variation of peak match running intensities in elite football. Biology of Sport, 39(4), 833-838. https://doi.org/10.5114/biolsport.2022.109456 |
| 1. Bok, D., Jukić, N., & Foster, C. (2022). Validation of session ratings of perceived exertion for quantifying training load in karate kata sessions. Biology of Sport, 39(4), 849-855. https://doi.org/10.5114/biolsport.2022.109458 |
| 1. Perez, J., Brocherie, F., Couturier, A., & Guilhem, G. (2022). International matches elicit stable mechanical workload in high-level female ice hockey. Biology of Sport, 39(4), 857-864. https://doi.org/10.5114/biolsport.2022.109455 |
| 1. Washif, J. A., Hébert-Losier, K., Chamari, K., & Beaven, C. M. (2022). Caffeine-carbohydrate mouth-rinsing counter-acts an observed negative effect of mouth-rinsing procedure during sprint-endurance training performance in fasted athletes: A pilot study. Biology of Sport, 39(4), 865-873. https://doi.org/10.5114/biolsport.2022.109959 |
| 1. González-Ravé, J., B. Pyne, D., Castillo, J. A., González-Mohíno, F., & Stone, M. H. (2022). Training periodization for a world-class 400 meters individual medley swimmer. Biology of Sport, 39(4), 883-888. https://doi.org/10.5114/biolsport.2022.109954 |
| 1. Akazawa, N., Ohiwa, N., Shimizu, K., Suzuki, N., Kumagai, H., & Fuku, N. et al. (2022). The association of ACTN3 R577X polymorphism with sports specificity in Japanese elite athletes. Biology of Sport, 39(4), 905-911. https://doi.org/10.5114/biolsport.2022.108704 |
| 1. Saito, M., Ginszt, M., Semenova, E. A., Massidda, M., Huminska-Lisowska, K., & Michałowska-Sawczyn, M. et al. (2022). Genetic profile of sports climbing athletes from three different ethnicities. Biology of Sport, 39(4), 913-919. https://doi.org/10.5114/biolsport.2022.109958 |
| 1. Guerrero-Calderón, B., Alfonso Morcillo, J., Chena, M., & Castillo-Rodríguez, A. (2022). Comparison of training and match load between metabolic and running speed metrics of professional Spanish soccer players by playing position. Biology of Sport, 39(4), 933-941. https://doi.org/10.5114/biolsport.2022.110884 |
| 1. Tayech, A., Arbi Mejri, M., Makhlouf, I., Uthof, A., Hambli, M., & G. Behm, D. et al. (2022). Reliability, criterion-concurrent validity, and construct-discriminant validity of a head-marking version of the taekwondo anaerobic intermittent kick test. Biology of Sport, 39(4), 951-963. https://doi.org/10.5114/biolsport.2022.109459 |
| 1. Thoseby, B., Govus, A. D., Clarke, A. C., Middleton, K. J., & Dascombe, B. J. (2022). Temporal distribution of peak running demands relative to match minutes in elite football. Biology of Sport, 39(4), 985-994. https://doi.org/10.5114/biolsport.2022.110745 |
| 1. Lahti, J., Mendiguchia, J., Edouard, P., & Morin, J. (2022). A novel multifactorial hamstring screening protocol: association with hamstring muscle injuries in professional football (soccer) – a prospective cohort study. Biology of Sport, 39(4), 1021-1031. https://doi.org/10.5114/biolsport.2022.112084 |
| 1. Romdhani, M., Souissi, N., Dergaa, I., Moussa-Chamari, I., Chaabouni, Y., & Mahdouani, K. et al. (2022). The effect of caffeine, nap opportunity and their combination on biomarkers of muscle damage and antioxidant defence during repeated sprint exercise. Biology of Sport, 39(4), 1033-1042. https://doi.org/10.5114/biolsport.2023.112088 |
| 1. Loturco, I., McGuigan, M. R., Freitas, T., Nakamura, F. Y., Boullosa, D. A., & Valenzuela, P. L. et al. (2022). Squat and countermovement jump performance across a range of loads: a comparison between Smith machine and free weight  execution modes in elite sprinters. Biology of Sport, 39(4), 1043-1048. https://doi.org/10.5114/biolsport.2022.112085 |
| 1. Matusiński, A., Gołas, A., Zajac, A., & Maszczyk, A. (2022). Acute effects of resisted and assisted locomotor activation on sprint performance. Biology of Sport, 39(4), 1049-1054. https://doi.org/10.5114/biolsport.2022.108706 |
| 1. Bortnik, L., Burger, J., & Rhodes, D. (2022). The mean and peak physical demands during transitional play and high pressure activities in elite football. Biology of Sport, 39(4), 1055-1064. https://doi.org/10.5114/biolsport.2023.112968 |
| 1. Li, Z., Mao, L., Krustrup, P., & Randers, M. (2022). Internal and external load during 8 v 8, 5 v 5 and 3 v 3 in Chinese elite youth male football players. Biology of Sport, 39(4), 1065-1071. https://doi.org/10.5114/biolsport.2022.113292 |
| 1. Washif, J. A., B. Pyne, D., Sandbakk, Ø., Trabelsi, K., Aziz, A., & Beaven, C. M. et al. (2022). Ramadan intermittent fasting induced poorer training practices during the COVID-19 lockdown: A global cross-sectional study with 5529 athletes from 110 countries. Biology of Sport, 39(4), 1103-1115. https://doi.org/10.5114/biolsport.2022.117576 |
| 1. Lee, M., Soo, J., Yeo, V., Rashid Aziz, A., & Ihsan, M. (2022). Running demands and activity profile of men’s rugby sevens: a tournament scenario. Biology of Sport, 39(3), 529-535. https://doi.org/10.5114/biolsport.2022.107023 |
| 1. Manzi, V., Annino, G., Savoia, C., Caminiti, G., Padua, E., & Masucci, M. et al. (2022). Relationship between aerobic fitness and metabolic power metrics in elite male soccer players. Biology of Sport, 39(3), 599-606. https://doi.org/10.5114/biolsport.2022.106389 |
| 1. Loturco, I., Pereira, L., Bishop, C., Zanetti, V., T. Freitas, T., & Pareja-Blanco, F. (2022). Effects of a resistance training intervention on the strength-deficit of elite young soccer players. Biology of Sport, 39(3), 615-619. https://doi.org/10.5114/biolsport.2022.106157 |
| 1. Vachon, A., Berryman, N., Mujika, I., Paquet, J., Sauvet, F., & Bosquet, L. (2022). Impact of tapering and proactive recovery on young elite rugby union players’ repeated high intensity effort ability. Biology of Sport, 39(3), 735-743. https://doi.org/10.5114/biolsport.2022.109453 |
| 1. Bachini, F.I., Pereira, D., Santos, R., Hausen, M., Pereira, G., & Vieira, C. et al. (2022). Creatine and creatinine quantification in olympic athletes: dried blood spot analysis pilot study. Biology of Sport, 39(3), 745-749. https://doi.org/10.5114/biolsport.2022.108701 |
| 1. Hew-Butler, T., Aprik, C., Byrd, B., Sabourin, J., VanSumeren, M., Smith-Hale, V., & Blow, A. (2022). Vitamin D supplementation and body composition changes in collegiate basketball players: a 12-week randomized control trial. Journal of the International Society of Sports Nutrition, 19(1), 34–48. https://doi.org/10.1080/15502783.2022.2046444 |
| 1. Hitendre, S., Jordan, R., Theodorakopoulos, C., & White, L. (2022). Dietary Intakes, Knowledge, and Perceptions of Semi-professional Rugby Athletes in Scotland. Journal of the International Society of Sports Nutrition, 19(1), 49–69. https://doi.org/10.1080/15502783.2022.2036436 |
| 1. Charlot, K., Lavoué, C., Siracusa, J., Chalchat, E., Hertert, P., & Bourrilhon, C. (2022). Fluctuations in food and fluid intake during a 24-h World Championship: analysis of the deviation from nutritional programs. Journal of the International Society of Sports Nutrition, 19(1), 92–109. https://doi.org/10.1080/15502783.2022.2046443 |
| 1. Jurov, I., Keay, N., & Rauter, S. (2022). Reducing energy availability in male endurance athletes: a randomized trial with a three-step energy reduction. Journal of the International Society of Sports Nutrition, 19(1), 179–195. https://doi.org/10.1080/15502783.2022.2065111 |
| 1. Ovchinnikov, A. N., Paoli, A., Seleznev, V. V., & Deryugina, A. V. (2022). Royal jelly plus coenzyme Q10 supplementation improves high-intensity interval exercise performance via changes in plasmatic and salivary biomarkers of oxidative stress and muscle damage in swimmers: a randomized, double-blind, placebo-controlled pilot trial. Journal of the International Society of Sports Nutrition, 19(1), 239–257. https://doi.org/10.1080/15502783.2022.2086015 |
| 1. Štangar, M., Štangar, A., Shtyrba, V., Cigić, B., & Benedik, E. (2022). Rapid weight loss among elite-level judo athletes: methods and nutrition in relation to competition performance. Journal of the International Society of Sports Nutrition, 19(1), 380–396. https://doi.org/10.1080/15502783.2022.2099231 |
| 1. Vogel, R. M., Ross, M. L., Swann, C., Rothwell, J. E., & Stevens, C. J. (2022). Athlete perceptions of flavored, menthol-enhanced energy gels ingested prior to endurance exercise in the heat. Journal of the International Society of Sports Nutrition, 19(1), 580–592. https://doi.org/10.1080/15502783.2022.2117995 |
| 1. Roklicer, R., Rossi, C., Bianco, A., Stajer, V., Ranisavljev, M., Todorovic, N., Manojlovic, M., Gilic, B., Trivic, T., & Drid, P. (2022). Prevalence of rapid weight loss in Olympic style wrestlers. Journal of the International Society of Sports Nutrition, 19(1), 593–602. https://doi.org/10.1080/15502783.2022.2119095 |
| 1. Juan J. Guerrero-Pinzón, Juan M.A. Alcantara, Gustavo García-Buendia, Sol Mochón-Benguigui, Mauricio Ramírez-Maldonado, Jonatan R. Ruiz & Lucas Jurado-Fasoli (2022) A nutritional intervention for moderate altitude endurance preparation: A case report, Journal of the International Society of Sports Nutrition, 19:1, 650-663 |
| 1. Jimenez, A. E., Owens, J. S., Monahan, P. F., Maldonado, D. R., Saks, B. R., Sabetian, P. W., Ankem, H. K., Lall, A. C., & Domb, B. G. (2022). Return to Sports and Minimum 2-Year Outcomes of Hip Arthroscopy in Elite Athletes With and Without Coexisting Low Back Pain: A Propensity-Matched Comparison. The American journal of sports medicine, 50(1), 68–78. https://doi.org/10.1177/03635465211056964 |
| 1. Chandran, A., Boltz, A. J., Morris, S. N., Robison, H. J., Nedimyer, A. K., Collins, C. L., & Register-Mihalik, J. K. (2022). Epidemiology of Concussions in National Collegiate Athletic Association (NCAA) Sports: 2014/15-2018/19. The American journal of sports medicine, 50(2), 526–536. https://doi.org/10.1177/03635465211060340 |
| 1. Manzi, J. E., Ciccotti, M. C., Trauger, N., Black, G. G., Thacher, R. R., Boddapati, V., & Dines, J. S. (2022). Increased Elbow and Olecranon Injury History in Professional Pitchers With Increased Elbow Flexion at Ball Release. The American journal of sports medicine, 50(4), 1054–1060. https://doi.org/10.1177/03635465211072223 |
| 1. Ferris, L. M., Kontos, A. P., Eagle, S. R., Elbin, R. J., Collins, M. W., Mucha, A., McAllister, T. W., Broglio, S. P., McCrea, M., Pasquina, P. F., & Port, N. L. (2022). Utility of VOMS, SCAT3, and ImPACT Baseline Evaluations for Acute Concussion Identification in Collegiate Athletes: Findings From the NCAA-DoD Concussion Assessment, Research and Education (CARE) Consortium. The American journal of sports medicine, 50(4), 1106–1119. https://doi.org/10.1177/03635465211072261 |
| 1. Lorentz, N. A., Hurley, E. T., Colasanti, C. A., Markus, D. H., Alaia, M. J., Campbell, K. A., Strauss, E. J., & Jazrawi, L. M. (2022). Return to Play After Biceps Tenodesis for Isolated SLAP Tears in Overhead Athletes. The American journal of sports medicine, 50(5), 1369–1374. https://doi.org/10.1177/03635465211041698 |
| 1. Waltz, R. A., Comfort, S. M., Pierpoint, L. A., Briggs, K. K., & Philippon, M. J. (2022). Femoroacetabular Impingement in Elite Skiers and Snowboarders: Return to Sports and Outcomes After Hip Arthroscopy. The American journal of sports medicine, 50(6), 1564–1570. https://doi.org/10.1177/03635465221085663 |
| 1. Guy, S., Fayard, J. M., Saithna, A., Bahroun, S., Ferreira, A., Carrozzo, A., De Jesus, S., Bulle, S., Vieira, T. D., & Sonnery-Cottet, B. (2022). Risk of Graft Rupture After Adding a Lateral Extra-articular Procedure at the Time of ACL Reconstruction: A Retrospective Comparative Study of Elite Alpine Skiers From the French National Team. The American journal of sports medicine, 50(6), 1609–1617. https://doi.org/10.1177/03635465221085027 |
| 1. Thompson, J. W., Rajput, V., Kayani, B., Plastow, R., Magan, A., & Haddad, F. S. (2022). Surgical Repair of Stener-like Injuries of the Medial Collateral Ligament of the Knee in Professional Athletes. The American journal of sports medicine, 50(7), 1815–1822. https://doi.org/10.1177/03635465221093807 |
| 1. Owens, J. S., Jimenez, A. E., Lee, M. S., Hawkins, G. C., Maldonado, D. R., & Domb, B. G. (2022). Basketball Players Undergoing Primary Hip Arthroscopy Exhibit Higher Grades of Acetabular Cartilage Damage but Achieve Favorable Midterm Outcomes and Return to Sports Rates Comparable With a Propensity-Matched Group of Other Cutting Sports Athletes. The American journal of sports medicine, 50(7), 1909–1918. https://doi.org/10.1177/03635465221092762 |
| 1. Manzi, J. E., Zeitlin, J. H., Dowling, B., Roberts, N., Ruzbarsky, J. J., Ciccotti, M. C., & Dines, J. S. (2022). Evaluating Pelvis Rotation Style at Foot Contact: A Propensity Scored Biomechanical Analysis in High School and Professional Pitchers. The American journal of sports medicine, 50(8), 2271–2280. https://doi.org/10.1177/03635465221094323 |
| 1. Knurr, K. A., Kliethermes, S. A., Haack, C. R., Olson, J. S., Binkley, N. C., Scerpella, T. A., & Heiderscheit, B. C. (2022). Changes in Bone Mineral Density of the Femur and Tibia Before Injury to 2 Years After Anterior Cruciate Ligament Reconstruction in Division I Collegiate Athletes. The American journal of sports medicine, 50(9), 2410–2416. https://doi.org/10.1177/03635465221099456 |
| 1. Harris, J., Maier, J., Freeston, J., Soloff, L., Himmerick, D., Pipkin, A., Genin, J. A., Schickendantz, M. S., & Frangiamore, S. J. (2022). Differences in Glenohumeral Range of Motion and Humeral Torsion Between Right-Handed and Left-Handed Professional Baseball Pitchers. The American journal of sports medicine, 50(9), 2481–2487. https://doi.org/10.1177/03635465221092115 |
| 1. Oldham, J. R., Lanois, C. J., Caccese, J. B., Crenshaw, J. R., Knight, C. A., Berkstresser, B., Wang, F., Howell, D. R., Meehan, W. P., 3rd, & Buckley, T. A. (2022). Association Between Collision Sport Career Duration and Gait Performance in Male Collegiate Student-Athletes. The American journal of sports medicine, 50(9), 2526–2533. https://doi.org/10.1177/03635465221104685 |
| 1. Jauhiainen, S., Kauppi, J. P., Krosshaug, T., Bahr, R., Bartsch, J., & Äyrämö, S. (2022). Predicting ACL Injury Using Machine Learning on Data From an Extensive Screening Test Battery of 880 Female Elite Athletes. The American journal of sports medicine, 50(11), 2917–2924. https://doi.org/10.1177/03635465221112095 |
| 1. Hopper, G. P., Pioger, C., Philippe, C., El Helou, A., Campos, J. P., Gousopoulos, L., Carrozzo, A., Vieira, T. D., & Sonnery-Cottet, B. (2022). Risk Factors for Anterior Cruciate Ligament Graft Failure in Professional Athletes: An Analysis of 342 Patients With a Mean Follow-up of 100 Months From the SANTI Study Group. The American journal of sports medicine, 50(12), 3218–3227. https://doi.org/10.1177/03635465221119186 |
| 1. Crotin, R. L., Slowik, J. S., Brewer, G., Cain, E. L., Jr, & Fleisig, G. S. (2022). Determinants of Biomechanical Efficiency in Collegiate and Professional Baseball Pitchers. The American journal of sports medicine, 50(12), 3374–3380. https://doi.org/10.1177/03635465221119194 |
| 1. Borque, K. A., Jones, M., Laughlin, M. S., Balendra, G., Willinger, L., Pinheiro, V. H., & Williams, A. (2022). Effect of Lateral Extra-articular Tenodesis on the Rate of Revision Anterior Cruciate Ligament Reconstruction in Elite Athletes. The American journal of sports medicine, 50(13), 3487–3492. https://doi.org/10.1177/03635465221128828 |
| 1. Owens, J. S., Lee, M. S., Jimenez, A. E., Maldonado, D. R., Lall, A. C., & Domb, B. G. (2022). Elite Female Athletes Demonstrate a Comparable Improvement in Midterm Patient-Reported Outcome Scores and Rate of Return to Sport Compared With Elite Male Athletes After Hip Arthroscopic Surgery: A Sex-Based Comparison in Professional and Collegiate Athletes. The American journal of sports medicine, 50(13), 3600–3609. https://doi.org/10.1177/03635465221123060 |
| 1. Pinheiro, V. H., Jones, M., Borque, K. A., Balendra, G., White, N. P., Ball, S. V., & Williams, A. (2022). Rates and Levels of Elite Sport Participation at 5 Years After Revision ACL Reconstruction. The American journal of sports medicine, 50(14), 3762–3769. https://doi.org/10.1177/03635465221127297 |
| 1. Susanne Ellens, Daniel Hodges, Sean McCullagh, James J. Malone & Matthew C. Varley (2022) Interchangeability of player movement variables from different athlete tracking systems in professional soccer, Science and Medicine in Football, 6:1, 1-6, |
| 1. Brian Doyle, Declan Browne & Dan Horan (2022) Quantification of internal and external training load during a training camp in senior international female footballers, Science and Medicine in Football, 6:1, 7-14, |
| 1. Afsaneh Safar Cherati, Salman Khalifeh Soltani, Navid Moghadam, Bahar Hassanmirzaei, Zohreh Haratian, Shayesteh Khalifeh Soltani & Meisam Rezaei (2022) Is there a relationship between lower-extremity injuries and foot postures in professional football players? A prospective cohort study, Science and Medicine in Football, 6:1, 49-59 |
| 1. Gibson Moreira Praça, André Gustavo Pereira Andrade, Sarah da Glória Teles Bredt, Felipe Arruda Moura & Pedro Emilio Drumond Moreira (2022) Progression to the target vs. regular rules in Soccer small-sided Games, Science and Medicine in Football, 6:1, 66-71, |
| 1. Jessica B. Farley, Carl T. Woods, Justin W. L. Keogh & Nikki Milne (2022) Profiling the kicking and handballing accuracy of female Australian football players across five competition levels, Science and Medicine in Football, 6:1, 72-81, |
| 1. Michèle Renard, Ana Anton-Solanas, David T. Kelly & Ciarán Ó Catháin (2022) Evaluation of nutrition knowledge in elite and sub-elite Gaelic football players, Science and Medicine in Football, 6:1, 82-88 |
| 1. Jessica B. Farley, Justin W. L. Keogh, Carl T. Woods & Nikki Milne (2022) Physical fitness profiles of female Australian football players across five competition levels, Science and Medicine in Football, 6:1, 105-126 |
| 1. Einari Kurittu, Tommi Vasankari, Tuomas Brinck, Jari Parkkari, Olli J. Heinonen, Pekka Kannus, Timo Hänninen, Klaus Köhler & Mari Leppänen (2022) Injury incidence and prevalence in Finnish top-level football – one-season prospective cohort study, Science and Medicine in Football, 6:2, 141-147, |
| 1. Rogério Ferreira Liporaci, Sergio Yoshimura & Bruno Manfredini Baroni (2022) Perceptions of Professional Football Players on Injury Risk Factors and Prevention Strategies, Science and Medicine in Football, 6:2, 148-152 |
| 1. Lloyd J. Parker, Kirsty J. Elliott-Sale, Marcus P. Hannon, James P. Morton & Graeme L. Close (2022) An audit of hormonal contraceptive use in Women’s Super League soccer players; implications on symptomology, Science and Medicine in Football, 6:2, 153-158 |
| 1. Lachlan Mitchell, Paul Lehane, Mark McCarthy, Kris O’Shea, Mark Tracey, Taylor Whyte & Ross Neville (2022) Nutrition knowledge of elite and non-elite Gaelic footballers, Science and Medicine in Football, 6:2, 159-163 |
| 1. William B. Sheehan, Rhys Tribolet, Mark L. Watsford, Andrew R. Novak, Michael Rennie & Job Fransen (2022) Tactical analysis of individual and team behaviour in professional Australian Football, Science and Medicine in Football, 6:2, 172-180 |
| 1. Emiel Schulze, Ross Julian & Tim Meyer (2022) Exploring Factors Related to Goal Scoring Opportunities in Professional Football, Science and Medicine in Football, 6:2, 181-188 |
| 1. Joshua Marris, Steve Barrett, Grant Abt & Chris Towlson (2022) Quantifying technical actions in professional soccer using foot-mounted inertial measurement units, Science and Medicine in Football, 6:2, 203-214, |
| 1. Andrew N. Guard, Kenneth McMillan & Niall G. MacFarlane (2022) The influence of relative playing area and player numerical imbalance on physical and perceptual demands in soccer small-sided game formats, Science and Medicine in Football, 6:2, 221-227 |
| 1. Andrew Vella, Anthea C Clarke, Thomas Kempton, Samuel Ryan, Jacob Holden & Aaron J. Coutts (2022) Technical involvements and pressure applied influence movement demands in elite Australian Football Match-play, Science and Medicine in Football, 6:2, 228-233 |
| 1. Alberto Fílter, Jesús Olivares Jabalera, Alejandro Molina-Molina, Luis Suárez-Arrones, José Robles-Rodríguez, Thomas Dos’Santos, Irineu Loturco, Bernardo Requena & Alfredo Santalla (2022) Effect of ball inclusion on jump performance in soccer players: a biomechanical approach, Science and Medicine in Football, 6:2, 241-247, |
| 1. Naomi Datson, Lorenzo Lolli, Barry Drust, Greg Atkinson, Matthew Weston & Warren Gregson (2022) Inter-methodological quantification of the target change for performance test outcomes relevant to elite female soccer players, Science and Medicine in Football, 6:2, 248-261, |
| 1. Thomas W. Kaminski, Sara P. D. Chrisman, Joseph Glutting, Victoria Wahlquist, Shawn Eagle, Margot Putukian, Ryan Tierney, Steven P. Broglio, Thomas W. McAllister, Michael A. McCrea, Paul F. Pasquina, Anthony P. Kontos & CARE Site Investigators (2022) Mechanisms of injury for concussions in collegiate soccer: an NCAA/DoD CARE consortium study, Science and Medicine in Football, 6:3, 325-330, |
| 1. Sebastian Harenberg, Zachary McCarver, Justin Worley, Dennis Murr, Justine Vosloo, Rumit Singh Kakar, Rob McCaffrey, Kim Dorsch & Oliver Höner (2022) The effectiveness of 3D multiple object tracking training on decision-making in soccer, Science and Medicine in Football, 6:3, 355-362, |
| 1. Paola Rodriguez-Giustiniani, Ian Rollo & Stuart D.R. Galloway (2022) A preliminary study of the reliability of soccer skill tests within a modified soccer match simulation protocol, Science and Medicine in Football, 6:3, 363-371 |
| 1. Matthew Andrew, Ryan W. O’Brien, Paul R. Ford & Joe Causer (2022) Developmental activities of professional male British rugby-league players versus controls, Science and Medicine in Football, 6:3, 381-388 |
| 1. Jon Larruskain, Jose A. Lekue, Imanol Martin-Garetxana, Irantzu Barrio, Alan McCall & Susana M. Gil (2022) Injuries are negatively associated with player progression in an elite football academy, Science and Medicine in Football, 6:4, 405-414 |
| 1. Vella, S., Bolling, C., Verhagen, E., & Moore, I. S. (2022). Perceiving, reporting and managing an injury - perspectives from national team football players, coaches, and health professionals. Science & medicine in football, 6(4), 421–433. https://doi.org/10.1080/24733938.2021.1985164 |
| 1. Jamie Salter, R. Cresswell & D. Forsdyke (2022) The impact of simulated soccer match-play on hip and hamstring strength in academy soccer players, Science and Medicine in Football, 6:4, 465-472 |
| 1. Oliver J Morgan, Barry Drust, Jack D Ade & Mark A. Robinson (2022) Change of direction frequency off the ball: new perspectives in elite youth soccer, Science and Medicine in Football, 6:4, 473-482 |
| 1. L. Moreira, L.F. Malloy-Diniz, G.S. Pinheiro & V.T. Costa (2022) Are there differences in the attention of elite football players concerning playing positions?, Science and Medicine in Football, 6:4, 494-502, |
| 1. Zouhaier Farhani, Raouf Hammami, Javier Gene-Morales, Sabri Gaied Chortane, Anissa Bouassida, Alvaro Juesas & Juan C. Colado (2022) Bout duration and number of players of soccer small-sided games affect perceived enjoyment, physiological responses, and technical-tactical performance, Science and Medicine in Football, 6:4, 503-510 |
| 1. Rhys Tribolet, William B. Sheehan, Andrew R. Novak, Mark L. Watsford & Job Fransen (2022) Factors associated with cooperative network connectedness in a professional Australian football small-sided game, Science and Medicine in Football, 6:4, 511-518 |
| 1. Ana Merayo, Jose Miguel Gallego, Oscar sans, Lluis Capdevila, Alex Iranzo, Dai Sugimoto & Gil Rodas (2022) Quantity and quality of sleep in young players of a professional football club, Science and Medicine in Football, 6:4, 539-544, |
| 1. Ivan Baptista, Andreas K. Winther, Dag Johansen, Morten B. Randers, Sigurd Pedersen & Svein Arne Pettersen (2022) The variability of physical match demands in elite women’s football, Science and Medicine in Football, 6:5, 559-565 |
| 1. Naomi Myhill, Dan Weaving, Steve Barrett, Ryan King & Stacey Emmonds (2022) A multi-club analysis of the locomotor training characteristics of elite female soccer players, Science and Medicine in Football, 6:5, 572-580 |
| 1. Phoebe Read, Ritan Mehta, Craig Rosenbloom, Elena Jobson & Katrine Okholm Kryger (2022) Elite female football players’ perception of the impact of their menstrual cycle stages on their football performance. A semi-structured interview-based study, Science and Medicine in Football, 6:5, 616-625, |
| 1. Nonhlanhla S. Mkumbuzi, Senanile B. Dlamini, Fidelis Chibhabha, Fredrick M. Govere & Lucinda Manda-Taylor (2022) The menstrual cycle and football: The experiences of African women football players, Science and Medicine in Football, 6:5, 626-632 |
| 1. Samuel J. McHaffie, Carl Langan-Evans, James C. Morehen, Juliette A. Strauss, José L. Areta, Christopher Rosimus, Martin Evans, Kirsty J. Elliott-Sale, Colum J. Cronin & James P. Morton (2022) Normalising the conversation: a qualitative analysis of player and stakeholder perceptions of menstrual health support within elite female soccer, Science and Medicine in Football, 6:5, 633-642 |
| 1. Lloyd J. F. Parker, Kirsty J. Elliott-Sale, Marcus Hannon, James P. Morton & Graeme L. Close (2022) Where do you go when your periods go?: A case-study examining secondary amenorrhea in a professional internationally-capped female soccer player through the lens of the sport nutritionist, Science and Medicine in Football, 6:5, 643-649 |
| 1. Carly Perry, Aiden J. Chauntry & Francesca M. Champ (2022) Elite female footballers in England: an exploration of mental ill-health and help-seeking intentions, Science and Medicine in Football, 6:5, 650-659, |
| 1. Kotone Hirose & Carla Meijen (2022) An exploration of elite Japanese female footballers’ acute cultural transition experiences in Europe, Science and Medicine in Football, 6:5, 660-667 |
| 1. Samuel J. McHaffie, Carl Langan-Evans, James C. Morehen, Juliette A. Strauss, José L. Areta, Christopher Rosimus, Martin Evans, Kirsty J. Elliott-Sale, Colum J. Cronin & James P. Morton (2022) Carbohydrate fear, skinfold targets and body image issues: a qualitative analysis of player and stakeholder perceptions of the nutrition culture within elite female soccer, Science and Medicine in Football, 6:5, 675-685, |
| 1. Horan, D., Blake, C., Hägglund, M., Kelly, S., Roe, M., & Delahunt, E. (2022). Injuries in elite-level women's football-a two-year prospective study in the Irish Women's National League. Scandinavian journal of medicine & science in sports, 32(1), 177–190. https://doi.org/10.1111/sms.14062 |
| 1. Köykkä, M., Laaksonen, M. S., Ihalainen, S., Ruotsalainen, K., & Linnamo, V. (2022). Performance-determining factors in biathlon prone shooting without physical stress. Scandinavian journal of medicine & science in sports, 32(2), 414–423. https://doi.org/10.1111/sms.14087 |
| 1. Pineda, R. C., Krampe, R. T., Vanlandewijck, Y., & Van Biesen, D. (2022). Cognitive-motor multitasking in athletes with and without intellectual impairment. Scandinavian journal of medicine & science in sports, 32(2), 424–434. https://doi.org/10.1111/sms.14088 |
| 1. Pueo, B., Tortosa-Martínez, J., Chirosa-Rios, L. J., & Manchado, C. (2022). Throwing performance by playing positions of male handball players during the European Championship 2020. Scandinavian journal of medicine & science in sports, 32(3), 588–597. https://doi.org/10.1111/sms.14100 |
| 1. Robin, M., Nordez, A., & Dorel, S. (2022). Analysis of elite road-cycling sprints in relation to maximal power-velocity-endurance profile: a longitudinal one-case study. Scandinavian journal of medicine & science in sports, 32(3), 598–611. https://doi.org/10.1111/sms.14103 |
| 1. Krustrup, P., Mohr, M., Nybo, L., Draganidis, D., Randers, M.B., Ermidis, G., Ørntoft, C., Røddik, L., Batsilas, D., Poulios, A., Ørtenblad, N., Loules, G., Deli, C.K., Batrakoulis, A., Nielsen, J.L., Jamurtas, A.Z. and Fatouros, I.G. (2022), Muscle metabolism and impaired sprint performance in an elite women’s football game. Scand J Med Sci Sports, 32: 27-38. |
| 1. Mohr, M., Fatouros, I. G., Jamurtas, A. Z., Draganidis, D., Thomassen, M., Ørntoft, C., Ermidis, G., Loules, G., Batsilas, D., Poulios, A., Papanikolaou, K., Randers, M. B., Krustrup, P., & Nybo, L. (2022). Skeletal muscle phenotype and game performance in elite women football players. Scandinavian journal of medicine & science in sports, 32 Suppl 1, 39–53. https://doi.org/10.1111/sms.14022 |
| 1. Pedersen, S., Welde, B., Sagelv, E. H., Heitmann, K. A., B Randers, M., Johansen, D., & Pettersen, S. A. (2022). Associations between maximal strength, sprint, and jump height and match physical performance in high-level female football players. Scandinavian journal of medicine & science in sports, 32 Suppl 1, 54–61. https://doi.org/10.1111/sms.14009 |
| 1. Oliveira, C. B., Marques, C., Abreu, R., Figueiredo, P., Calhau, C., Brito, J., & Sousa, M. (2022). Gut microbiota of elite female football players is not altered during an official international tournament. Scandinavian journal of medicine & science in sports, 32 Suppl 1, 62–72. https://doi.org/10.1111/sms.14096 |
| 1. Oliveira, C. B., Sousa, M., Abreu, R., Ferreira, Â., Figueiredo, P., Rago, V., Teixeira, V. H., & Brito, J. (2022). Dietary supplements usage by elite female football players: an exploration of current practices. Scandinavian journal of medicine & science in sports, 32 Suppl 1, 73–80. https://doi.org/10.1111/sms.14001 |
| 1. Winther, A. K., Baptista, I., Pedersen, S., Randers, M. B., Johansen, D., Krustrup, P., & Pettersen, S. A. (2022). Position specific physical performance and running intensity fluctuations in elite women's football. Scandinavian journal of medicine & science in sports, 32 Suppl 1, 105–114. https://doi.org/10.1111/sms.14105 |
| 1. Panduro, J., Ermidis, G., Røddik, L., Vigh-Larsen, J. F., Madsen, E. E., Larsen, M. N., Pettersen, S. A., Krustrup, P., & Randers, M. B. (2022). Physical performance and loading for six playing positions in elite female football: full-game, end-game, and peak periods. Scandinavian journal of medicine & science in sports, 32 Suppl 1, 115–126. https://doi.org/10.1111/sms.13877 |
| 1. Póvoas, S., Ascensão, A., Magalhães, J., Silva, P., Wiig, H., Raastad, T., Castagna, C., & Andersson, H. (2022). Technical match actions and plasma stress markers in elite female football players during an official FIFA Tournament. Scandinavian journal of medicine & science in sports, 32 Suppl 1, 127–139. https://doi.org/10.1111/sms.13878 |
| 1. Costa, J. A., Brito, J., Nakamura, F. Y., Dores, H., & Rebelo, A. (2022). Associations between 24-h heart rate variability and aerobic fitness in high-level female soccer players. Scandinavian journal of medicine & science in sports, 32 Suppl 1, 140–149. https://doi.org/10.1111/sms.14116 |
| 1. Madsen, E. E., Hansen, T., Thomsen, S. D., Panduro, J., Ermidis, G., Krustrup, P., Randers, M. B., Larsen, C. H., Elbe, A. M., & Wikman, J. (2022). Can psychological characteristics, football experience, and player status predict state anxiety before important matches in Danish elite-level female football players?. Scandinavian journal of medicine & science in sports, 32 Suppl 1, 150–160. https://doi.org/10.1111/sms.13881 |
| 1. Holt, A. C., Siegel, R., Ball, K., Hopkins, W. G., & Aughey, R. J. (2022). Prediction of 2000-m on-water rowing performance with measures derived from instrumented boats. Scandinavian journal of medicine & science in sports, 32(4), 710–719. https://doi.org/10.1111/sms.14125 |
| 1. Merle, C. L., Richter, L., Challakh, N., Haak, R., Schmalz, G., Needleman, I., Wolfarth, B., Ziebolz, D., & Wüstenfeld, J. (2022). Orofacial conditions and oral health behavior of young athletes: A comparison of amateur and competitive sports. Scandinavian journal of medicine & science in sports, 32(5), 903–912. https://doi.org/10.1111/sms.14143 |
| 1. Carolan, D., Richter, C., Thorborg, K., Franklyn-Miller, A., O' Donovan, J., McDonald, C., & King, E. (2022). Hip and groin pain prevalence and prediction in Elite Gaelic Games: 2703 male athletes across two seasons. Scandinavian journal of medicine & science in sports, 32(5), 924–932. https://doi.org/10.1111/sms.14136 |
| 1. Rønnestad, B. R., Lid, O. M., Hansen, J., Hamarsland, H., Mølmen, K. S., Nygaard, H., Ellefsen, S., Hammarström, D., & Lundby, C. (2022). Heat suit training increases hemoglobin mass in elite cross-country skiers. Scandinavian journal of medicine & science in sports, 32(7), 1089–1098. https://doi.org/10.1111/sms.14156 |
| 1. Skattebo, Ø., & Hallén, J. (2022). Individual variations in pre-altitude hemoglobin mass influence hemoglobin mass responses to repeated altitude sojourns. Scandinavian journal of medicine & science in sports, 32(10), 1493–1501. https://doi.org/10.1111/sms.14218 |
| 1. Whiteley, R., Gregson, W., Bahr, R., Tabben, M., Chamari, K., Lolli, L., & Salvo, V. D. (2022). High-speed running during match-play before and after return from hamstring injury in professional footballers. Scandinavian journal of medicine & science in sports, 32(10), 1502–1509. https://doi.org/10.1111/sms.14219 |
| 1. Robertson, C. M., Williams, S., West, S. W., Starling, L., Kemp, S., Cross, M., & Stokes, K. A. (2022). Influence of playing surface on match injury risk in men's professional rugby union in England (2013-2019). Scandinavian journal of medicine & science in sports, 32(11), 1615–1624. https://doi.org/10.1111/sms.14226. |
| 1. Washino, S., Murai, A., Mankyu, H., Ogita, F., Kanehisa, H., & Yoshitake, Y. (2022). Projected frontal area and its components during front crawl depend on lung volume. Scandinavian journal of medicine & science in sports, 32(12), 1724–1737. https://doi.org/10.1111/sms.14231 |
| 1. Gallo, G., Mateo-March, M., Gotti, D., Faelli, E., Ruggeri, P., Codella, R., & Filipas, L. (2022). How do world class top 5 Giro d'Italia finishers train? A qualitative multiple case study. Scandinavian journal of medicine & science in sports, 32(12), 1738–1746. https://doi.org/10.1111/sms.14201 |
| 1. Atcharat Yongtawee, Jinhan Park, Yujin Kim & Minjung Woo (2022). Athletes have different dominant cognitive functions depending on type of sport, International Journal of Sport and Exercise Psychology, 20:1, 1-15, |
| 1. Selenia di Fronso, Sergio Costa, Cristina Montesano, Francesco Di Gruttola, Edoardo Giorgio Ciofi, Luana Morgilli, Claudio Robazza & Maurizio Bertollo (2022) The effects of COVID-19 pandemic on perceived stress and psychobiosocial states in Italian athletes, International Journal of Sport and Exercise Psychology, 20:1, 79-91 |
| 1. Jennifer Savage, Dave Collins & Andrew Cruickshank (2022) Perspective, control, and confidence: perceived outcomes of using psycho-behavioural skills in the developmental trauma experience, International Journal of Sport and Exercise Psychology, 20:2, 377-396 |
| 1. Martin Daumiller, Raven Rinas & Jennifer Breithecker (2022) Elite athletes’ achievement goals, burnout levels, psychosomatic stress symptoms, and coping strategies, International Journal of Sport and Exercise Psychology, 20:2, 416-435 |
| 1. Katherine V. Sparks, Maria Kavussanu, Rich S.W. Masters & Christopher Ring (2022) Conscious processing and rowing: a field study, International Journal of Sport and Exercise Psychology, 20:2, 515-531 |
| 1. Doug Crowell & Daniel J. Madigan (2022) Perfectionistic concerns cognitions predict burnout in college athletes: a three-month longitudinal study, International Journal of Sport and Exercise Psychology, 20:2, 532-550 |
| 1. Zeljka Vidic & Nicholas P. Cherup (2022) Take me into the ball game: an examination of a brief psychological skills training and mindfulness-based intervention with baseball players, International Journal of Sport and Exercise Psychology, 20:2, 612-629 |
| 1. Amin Daneshfar, Carl J. Petersen & Daniel E. Gahreman (2022) The effect of 4 weeks motor imagery training on simulated BMX race performance, International Journal of Sport and Exercise Psychology, 20:2, 644-660 |
| 1. Young-Eun Noh & Syazana Shahdan (2022) A model for formulating the relationship between religion and sport performance: a grounded theory approach, International Journal of Sport and Exercise Psychology, 20:3, 661-676 |
| 1. Ying-Lien Ni, Shih-Chi Hsu, Che-Chun Kuo, Mei-Yen Chen & Lung Hung Chen (2022) High income but high stress: cross over effects of work and family role conflict in professional athletes and their partners, International Journal of Sport and Exercise Psychology, 20:3, 677-697 |
| 1. Christie S. Y. Han, Renée L. Parsons-Smith, Gerard J. Fogarty & Peter C. Terry (2022) Psychometric properties of the Brunel Mood Scale among athletes and non-athletes in Singapore, International Journal of Sport and Exercise Psychology, 20:3, 698-714, |
| 1. Benjamin Hardwick, Daniel J. Madigan, Andrew P. Hill, Simon Kumar & Derwin K. C. Chan (2022) Perfectionism and attitudes towards doping in athletes: the mediating role of achievement goal orientations, International Journal of Sport and Exercise Psychology, 20:3, 743-756, |
| 1. Sara Oliveira, António Rosado, Marina Cunha & Cláudia Ferreira (2022) The compassionate coach scale as perceived by the athlete: development and initial validation in Portuguese athletes, International Journal of Sport and Exercise Psychology, 20:3, 794-812, |
| 1. Rosie Collins, Dave Collins & Howie J. Carson (2022) Muscular collision chess: a qualitative exploration of the role and development of cognition, understanding and knowledge in elite-level decision making, International Journal of Sport and Exercise Psychology, 20:3, 828-848 |
| 1. Marion Geary, Mark Campbell, Niamh Kitching & Frank Houghton (2022) “I’m a hurler … basically just a hurler”: a mixed methods study of the athletic identity of elite Irish Gaelic Athletic Association dual career athletes, International Journal of Sport and Exercise Psychology, 20:3, 872-895 |
| 1. Cailie S. McGuire, M. Blair Evans & Luc J. Martin (2022) Perceiving and experiencing subgroups in sport: a proposed conceptual framework, International Journal of Sport and Exercise Psychology, 20:3, 915-935, |
| 1. Charlotte Downing, Karin Redelius & Sanna Nordin-Bates (2022) Early specialisation among Swedish aesthetic performers: exploring motivation and perceptions of parental influence, International Journal of Sport and Exercise Psychology, 20:4, 1013-1032 |
| 1. Hatem Ben Mahfoudh & Bachir Zoudji (2022) The role of visuospatial abilities and the level of expertise in memorising soccer animations, International Journal of Sport and Exercise Psychology, 20:4, 1033-1048, |
| 1. Sofie Morbée, Leen Haerens, Joachim Waterschoot & Maarten Vansteenkiste (2022) Which cyclists manage to cope with the corona crisis in a resilient way? The role of motivational profiles, International Journal of Sport and Exercise Psychology, 20:4, 1049-1067 |
| 1. Vassilis Barkoukis, Michael Petrou, Lambros Lazuras & Despoina Ourda (2022) An empirical investigation of sport stakeholders’ beliefs about whistleblowing against doping behaviour, International Journal of Sport and Exercise Psychology, 20:4, 1068-1085, |
| 1. Michael Petrou, Lambros Lazuras, Mathew Hillier & Dara Mojtahedi (2022) Doping behaviour in mixed martial arts athletes: the roles of social norms and self-regulatory efficacy, International Journal of Sport and Exercise Psychology, 20:4, 1086-1101 |
| 1. Cristiano da Conceição Ferreira Murta, Maicon Rodrigues Albuquerque, Pablo Juan Greco, Markus Raab & Gibson Moreira Praça (2022) Goalkeepers put their money where the coach’s mouth is: Knowing kickers’ preferences enhances anticipation of football goalkeepers, International Journal of Sport and Exercise Psychology, 20:5, 1507-1522 |
| 1. Ronnie Lidor, Lior Lipshits, Michal Arnon & Michael Bar-Eli (2022) “Don't think, just shoot” – The paradox of shooting three-point shots in basketball, International Journal of Sport and Exercise Psychology, 20:6, 1523-1541 |
| 1. Siobhan Henderson, Gordon A. Bloom & Danielle Alexander (2022) Desired coaching behaviours of elite divers during competition, International Journal of Sport and Exercise Psychology, 20:6, 1777-1794 |
| 1. Lis, D. M., Jordan, M., Lipuma, T., Smith, T., Schaal, K., & Baar, K. (2022). Collagen and Vitamin C Supplementation Increases Lower Limb Rate of Force Development. International Journal of Sport Nutrition and Exercise Metabolism, 32(2), 65-73. |
| 1. Ferguson, C., Aisbett, B., Lastella, M., Roberts, S., & Condo, D. (2022). Evening Whey Protein Intake, Rich in Tryptophan, and Sleep in Elite Male Australian Rules Football Players on Training and Nontraining Days. International Journal of Sport Nutrition and Exercise Metabolism, 32(2), 82-88 |
| 1. Fensham, N. C., McKay, A. K., Tee, N., Lundy, B., Anderson, B., Morabito, A., Ross, M. L., & Burke, L. M. (2022). Sequential Submaximal Training in Elite Male Rowers Does Not Result in Amplified Increases in Interleukin-6 or Hepcidin. International Journal of Sport Nutrition and Exercise Metabolism, 32(3), 177-185 |
| 1. Graybeal, A. J., Helms, B., Couris, K., Thomas, D., Johnston, T., Dahan, V., Escobedo, N., & Willis, J. L. (2022). Improved Physiological Markers of Omega-3 Status and Compliance With Omega-3 Supplementation in Division I Track and Field and Cross-Country Athletes: A Randomized Controlled Crossover Trial. International Journal of Sport Nutrition and Exercise Metabolism, 32(4), 246-255. |
| 1. Peacock, O. J., Gonzalez, J. T., Roberts, S. P., Smith, A., Drawer, S., & Stokes, K. A. (2022). Ketone Monoester Ingestion Alters Metabolism and Simulated Rugby Performance in Professional Players. International Journal of Sport Nutrition and Exercise Metabolism, 32(5), 334-341. |
| 1. Baker, L. B., King, M. A., Keyes, D. M., Brown, S. D., Engel, M. D., Seib, M. S., Aranyosi, A. J., & Ghaffari, R. (2022). Sweating Rate and Sweat Chloride Concentration of Elite Male Basketball Players Measured With a Wearable Microfluidic Device Versus the Standard Absorbent Patch Method. International Journal of Sport Nutrition and Exercise Metabolism, 32(5), 342-349 |
| 1. Sitko, S., Cirer-Sastre, R., Corbi, F., & López-Laval, I. (2022). Five-Minute Power-Based Test to Predict Maximal Oxygen Consumption in Road Cycling. International Journal of Sports Physiology and Performance, 17(1), 9-15. |
| 1. Koyama, T., Rikukawa, A., Nagano, Y., Sasaki, S., Ichikawa, H., & Hirose, N. (2022). High-Acceleration Movement, Muscle Damage, and Perceived Exertion in Basketball Games. International Journal of Sports Physiology and Performance, 17(1), 16-21. |
| 1. Muriel, X., Valenzuela, P. L., Mateo-March, M., Pallarés, J. G., Lucia, A., & Barranco-Gil, D. (2022). Physical Demands and Performance Indicators in Male Professional Cyclists During a Grand Tour: WorldTour Versus ProTeam Category. International Journal of Sports Physiology and Performance, 17(1), 22-30. |
| 1. Clancy, C., Gleeson, N., & Mercer, T. (2022). Neuromuscular Performance and Training Workload Over an In-Season Mesocycle in Elite Young Soccer Players. International Journal of Sports Physiology and Performance, 17(1), 37-43 |
| 1. Jones, T. W., Govus, A. D., Buskqvist, A., Andersson, E. P., & McGawley, K. (2022). An Analysis of Warm-Up Strategies at a Cross-Country Skiing National Championship. International Journal of Sports Physiology and Performance, 17(1), 50-57 |
| 1. Koral, J., Fanget, M., Imbert, L., Besson, T., Kennouche, D., Parent, A., Foschia, C., Rossi, J., & Millet, G. Y. (2022). Fatigue Measured in Dynamic Versus Isometric Modes After Trail Running Races of Various Distances. International Journal of Sports Physiology and Performance, 17(1), 67-77 |
| 1. Haischer, M. H., Krzyszkowski, J., Roche, S., & Kipp, K. (2022). Maximal Strength in Relation to Force and Velocity Patterns During Countermovement Jumps. International Journal of Sports Physiology and Performance, 17(1), 83-89 |
| 1. Rønnestad, B. R., Hansen, J., Bonne, T. C., & Lundby, C. (2022). Case Report: Heat Suit Training May Increase Hemoglobin Mass in Elite Athletes. International Journal of Sports Physiology and Performance, 17(1), 115-119. |
| 1. Caia, J., Halson, S. L., Holmberg, P. M., & Kelly, V. G. (2022). Does Caffeine Consumption Influence Postcompetition Sleep in Professional Rugby League Athletes? A Case Study. International Journal of Sports Physiology and Performance, 17(1), 126-129 |
| 1. Zacca, R., Mezêncio, B., de Souza Castro, F. A., Nakamura, F. Y., Pyne, D. B., Vilas-Boas, J. P., & Fernandes, R. J. (2022). Case Study: Comparison of Swimsuits and Wetsuits Through Biomechanics and Energetics in Elite Female Open Water Swimmers. International Journal of Sports Physiology and Performance, 17(1), 130-136. |
| 1. Kenneally, M., Casado, A., Gomez-Ezeiza, J., & Santos-Concejero, J. (2022). Training Characteristics of a World Championship 5000-m Finalist and Multiple Continental Record Holder Over the Year Leading to a World Championship Final. International Journal of Sports Physiology and Performance, 17(1), 142-146 |
| 1. Russell, S., Jenkins, D. G., Halson, S. L., Juliff, L. E., Connick, M. J., & Kelly, V. G. (2022). Mental Fatigue Over 2 Elite Netball Seasons: A Case for Mental Fatigue to be Included in Athlete Self-Report Measures. International Journal of Sports Physiology and Performance, 17(2), 160-169. |
| 1. Bartram, J. C., Thewlis, D., Martin, D. T., & Norton, K. I. (2022). Validating an Adjustment to the Intermittent Critical Power Model for Elite Cyclists—Modeling W′ Balance During World Cup Team Pursuit Performances. International Journal of Sports Physiology and Performance, 17(2), 170-175 |
| 1. Vajda, M., & Piatrikova, E. (2022). Relationship Between Flat-Water Tests and Canoe Slalom Performance on 4 Different Grades of Water Terrain Difficulty. International Journal of Sports Physiology and Performance, 17(2), 185-194 |
| 1. Halson, S. L., Appaneal, R. N., Welvaert, M., Maniar, N., & Drew, M. K. (2022). Stressed and Not Sleeping: Poor Sleep and Psychological Stress in Elite Athletes Prior to the Rio 2016 Olympic Games. International Journal of Sports Physiology and Performance, 17(2), 195-202. |
| 1. van Erp, T., Lamberts, R. P., & Sanders, D. (2022). Power Profile of Top 5 Results in World Tour Cycling Races. International Journal of Sports Physiology and Performance, 17(2), 203-209. |
| 1. van Erp, T., van der Hoorn, T., Hoozemans, M. J., Foster, C., & de Koning, J. J. (2022). Various Workload Models and the Preseason Are Associated With Injuries in Professional Female Cyclists. International Journal of Sports Physiology and Performance, 17(2), 210-215. |
| 1. Miles, K. H., Clark, B., Mara, J. K., Fowler, P. M., Miller, J., & Pumpa, K. L. (2022). How Do the Habitual Sleep Patterns of Elite Female Basketball and Soccer Athletes Compare With the General Population?. International Journal of Sports Physiology and Performance, 17(2), 234-240 |
| 1. Rago, V., Muschinsky, A., Deylami, K., Mohr, M., & Vigh-Larsen, J. F. (2022). Weekly Training Load in Elite Male Ice Hockey: Practice Versus Competition Demands. International Journal of Sports Physiology and Performance, 17(2), 270-277. |
| 1. Redman, K. J., Wade, L., Kelly, V. G., Connick, M. J., & Beckman, E. M. (2022). Predicting Rugby League Tackle Outcomes Using Strength and Power Principal Components. International Journal of Sports Physiology and Performance, 17(2), 278-285 |
| 1. Schofield, K. L., Thorpe, H., & Sims, S. T. (2022). Case Study: Energy Availability and Endocrine Markers in Elite Male Track Cyclists. International Journal of Sports Physiology and Performance, 17(2), 313-316. |
| 1. Ohya, T., Kusanagi, K., Koizumi, J., Ando, R., Katayama, K., & Suzuki, Y. (2022). Effect of Moderate- or High-Intensity Inspiratory Muscle Strength Training on Maximal Inspiratory Mouth Pressure and Swimming Performance in Highly Trained Competitive Swimmers. International Journal of Sports Physiology and Performance, 17(3), 343-349 |
| 1. Baiget, E., Colomar, J., & Corbi, F. (2022). Upper-Limb Force–Time Characteristics Determine Serve Velocity in Competition Tennis Players. International Journal of Sports Physiology and Performance, 17(3), 358-366 |
| 1. Ribeiro, J. N., Monteiro, D., Gonçalves, B., Brito, J., Sampaio, J., & Travassos, B. (2022). Variation in Physical Performance of Futsal Players During Congested Fixtures. International Journal of Sports Physiology and Performance, 17(3), 367-373. |
| 1. Rønnestad, B. R., Bakken, T. A., Thyli, V., Hansen, J., Ellefsen, S., & Hammarstrøm, D. (2022). Increasing Oxygen Uptake in Cross-Country Skiers by Speed Variation in Work Intervals. International Journal of Sports Physiology and Performance, 17(3), 384-390. |
| 1. Neumann, N. D., Van Yperen, N. W., Brauers, J. J., Frencken, W., Brink, M. S., Lemmink, K. A., Meerhoff, L. A., & Den Hartigh, R. J. (2022). Nonergodicity in Load and Recovery: Group Results Do Not Generalize to Individuals. International Journal of Sports Physiology and Performance, 17(3), 391-399 |
| 1. Partridge, E. M., Cooke, J., McKune, A. J., & Pyne, D. B. (2022). Partial-Body Cryotherapy Exposure 2 Hours Prior to a Shuttle Run Does Not Enhance Running Performance. International Journal of Sports Physiology and Performance, 17(3), 415-422 |
| 1. Sherman, S. R., Holmes, C. J., Demos, A. P., Stone, T., Hornikel, B., MacDonald, H. V., Fedewa, M. V., & Esco, M. R. (2022). Vagally Derived Heart Rate Variability and Training Perturbations With Menses in Female Collegiate Rowers. International Journal of Sports Physiology and Performance, 17(3), 432-439 |
| 1. O’Brien, T. J., Briley, S. J., Mason, B. S., Leicht, C. A., Tolfrey, K., & Goosey-Tolfrey, V. L. (2022). A High-Intensity Warm-Up Increases Thermal Strain But Does Not Affect Repeated Sprint Performance in Athletes With a Cervical Spinal Cord Injury. International Journal of Sports Physiology and Performance, 17(3), 440-449. |
| 1. Gallo, G., Leo, P., Mateo-March, M., Giorgi, A., Faelli, E., Ruggeri, P., Mujika, I., & Filipas, L. (2022). Cross-Sectional Differences in Race Demands Between Junior, Under 23, and Professional Road Cyclists. International Journal of Sports Physiology and Performance, 17(3), 450-457. |
| 1. Pareja-Blanco, F., Pereira, L. A., Reis, V. P., Fernandes, V., Arruda, A. F., Guerriero, A., Alcaraz, P. E., Freitas, T. T., & Loturco, I. (2022). Impact of Sled Loads on Performance and Kinematics of Elite Sprinters and Rugby Players. International Journal of Sports Physiology and Performance, 17(3), 465-473 |
| 1. Staunton, C. A., Swarén, M., Stöggl, T., Born, D., & Björklund, G. (2022). The Relationship Between Cardiorespiratory and Accelerometer-Derived Measures in Trail Running and the Influence of Sensor Location. International Journal of Sports Physiology and Performance, 17(3), 474-483. |
| 1. Loureiro, L. L., Ferreira, T. J., da Costa, C. S., Fidalgo, T. K., Valente, A. P., & Pierucci, A. P. T. (2022). Impact of Precompetitive Training on Metabolites in Modern Pentathletes. International Journal of Sports Physiology and Performance, 17(3), 489-494 |
| 1. Sargent, C., Halson, S. L., Martin, D. T., & Roach, G. D. (2022). Consecutive Days of Racing Does Not Affect Sleep in Professional Road Cyclists. International Journal of Sports Physiology and Performance, 17(3), 495-498 |
| 1. Gattoni, C., Girardi, M., O’Neill, B. V., & Maria Marcora, S. (2022). Sleep Deprivation Training to Reduce the Negative Effects of Sleep Loss on Endurance Performance: A Single Case Study. International Journal of Sports Physiology and Performance, 17(3), 499-503. |
| 1. Teixeira, F. G., Rosa, P. T. C. R., Mello, R. G. T., & Nadal, J. (2022). Biomechanical and Anthropometric Factors That Differentiate National- and Regional-Level Judo Players. International Journal of Sports Physiology and Performance, 17(4), 523-529 |
| 1. Debien, P. B., Timoteo, T. F., Gabbett, T. J., & Bara Filho, M. G. (2022). Training-Load Management in Rhythmic Gymnastics: Practices and Perceptions of Coaches, Medical Staff, and Gymnasts. International Journal of Sports Physiology and Performance, 17(4), 530-540 |
| 1. Gillam, I. H., Cunningham, R. B., & Telford, R. D. (2022). Antioxidant Supplementation Protects Elite Athlete Muscle Integrity During Submaximal Training. International Journal of Sports Physiology and Performance, 17(4), 549-555 |
| 1. Vitale, J. A., Galbiati, A., De Giacomi, G., Tornese, D., Levendowski, D., Ferini-Strambi, L., & Banfi, G. (2022). Sleep Architecture in Response to a Late Evening Competition in Team-Sport Athletes. International Journal of Sports Physiology and Performance, 17(4), 569-575. |
| 1. Komka, Z., Szilágyi, B., Molnár, D., Sipos, B., Tóth, M., Elek, J., & Szász, M. (2022). High-Resolution Dynamics of Hemodilution After Exercise-Related Hemoconcentration. International Journal of Sports Physiology and Performance, 17(4), 576-585. |
| 1. Berriel, G. P., Cardoso, A. S., Costa, R. R., Rosa, R. G., Oliveira, H. B., Kruel, L. F. M., & Peyré-Tartaruga, L. A. (2022). Does Complex Training Enhance Vertical Jump Performance and Muscle Power in Elite Male Volleyball Players?. International Journal of Sports Physiology and Performance, 17(4), 586-593 |
| 1. Saidi, K., Zouhal, H., Boullosa, D., Dupont, G., Hackney, A. C., Bideau, B., Granacher, U., & Ben Abderrahman, A. (2022). Biochemical Markers and Wellness Status During a Congested Match Play Period in Elite Soccer Players. International Journal of Sports Physiology and Performance, 17(4), 605-620 |
| 1. Conte, D., Arruda, A. F. S., Clemente, F. M., Castillo, D., Kamarauskas, P., & Guerriero, A. (2022). Assessing the Relationship Between External and Internal Match Loads in Elite Women’s Rugby Sevens. International Journal of Sports Physiology and Performance, 17(4), 634-639 |
| 1. Gielen, J., Mehuys, E., Berckmans, D., Meeusen, R., & Aerts, J. (2022). Monitoring Internal and External Load During Volleyball Competition. International Journal of Sports Physiology and Performance, 17(4), 640-645. |
| 1. Besson, C., Guex, K., Schmitt, L., Gojanovic, B., & Gremeaux, V. (2022). Successful Return to Performance After COVID-19 Infection in an Elite Athlete. International Journal of Sports Physiology and Performance, 17(4), 667-670 |
| 1. Mateo-March, M., van Erp, T., Muriel, X., Valenzuela, P. L., Zabala, M., Lamberts, R. P., Lucia, A., Barranco-Gil, D., & Pallarés, J. G. (2022). The Record Power Profile in Professional Female Cyclists: Normative Values Obtained From a Large Database. International Journal of Sports Physiology and Performance, 17(5), 682-686. |
| 1. Fessl, I., Wiesinger, H., & Kröll, J. (2022). Power–Force–Velocity Profiling as a Function of Used Loads and Task Experience. International Journal of Sports Physiology and Performance, 17(5), 694-700. |
| 1. Valenzuela, P. L., Muriel, X., van Erp, T., Mateo-March, M., Gandia-Soriano, A., Zabala, M., Lamberts, R. P., Lucia, A., Barranco-Gil, D., & Pallarés, J. G. (2022). The Record Power Profile of Male Professional Cyclists: Normative Values Obtained From a Large Database. International Journal of Sports Physiology and Performance, 17(5), 701-710. |
| 1. Ruggiero, L., Pritchard, S. E., Warmenhoven, J., Bruce, T., MacDonald, K., Klimstra, M., & McNeil, C. J. (2022). Volleyball Competition on Consecutive Days Modifies Jump Kinetics but Not Height. International Journal of Sports Physiology and Performance, 17(5), 711-719 |
| 1. Kanthack, T. F. D., Guillot, A., Simon, I., & Di Rienzo, F. (2022). Evaluating the Effect of the Combat Style Congruency on Performance and Fatigability in Brazilian Jiu-Jitsu: A Pilot Study. International Journal of Sports Physiology and Performance, 17(5), 726-732. |
| 1. Redman, K. J., Wade, L., Kelly, V. G., Connick, M. J., & Beckman, E. M. (2022). Effects of the Off-Season on Muscular Power in Professional Rugby League. International Journal of Sports Physiology and Performance, 17(5), 733-738 |
| 1. Eggers, T., Cross, R., Norris, D., Wilmot, L., & Lovell, R. (2022). Impact of Microcycle Structures on Physical and Technical Outcomes During Professional Rugby League Training and Matches. International Journal of Sports Physiology and Performance, 17(5), 755-760 |
| 1. Howe, S. T., Aughey, R. J., Hopkins, W. G., & Stewart, A. M. (2022). Modeling Professional Rugby Union Peak Intensity–Duration Relationships Using a Power Law. International Journal of Sports Physiology and Performance, 17(5), 780-786 |
| 1. Freitas, T. T., Alcaraz, P. E., Winckler, C., Zabaloy, S., Pereira, L. A., & Loturco, I. (2022). Differences in Strength, Speed, and Power Performance Between Visually Impaired Paralympic and Olympic Sprinters. International Journal of Sports Physiology and Performance, 17(5), 787-790 |
| 1. Vachon, A., Berryman, N., Mujika, I., Paquet, J., & Bosquet, L. (2022). Preconditioning Activities to Enhance Repeated High-Intensity Efforts in Elite Rugby Union Players. International Journal of Sports Physiology and Performance, 17(6), 871-878. |
| 1. Letter, R. T., Dwyer, D. B., Drinkwater, E. J., & Feros, S. A. (2022). The Physical Differences Between Faster and Slower Elite Male and Female Pace Bowlers. International Journal of Sports Physiology and Performance, 17(6), 879-885. |
| 1. Mateo-March, M., Valenzuela, P. L., Muriel, X., Gandia-Soriano, A., Zabala, M., Lucia, A., Pallares, J. G., & Barranco-Gil, D. (2022). The Record Power Profile of Male Professional Cyclists: Fatigue Matters. International Journal of Sports Physiology and Performance, 17(6), 926-931. |
| 1. Sargent, C., Rogalski, B., Montero, A., & Roach, G. D. (2022). The Sleep Behaviors of Elite Australian Rules Footballers Before and After Games During an Entire Season. International Journal of Sports Physiology and Performance, 17(6), 932-942 |
| 1. Yang, W., Park, J., Shin, Y., & Kim, J. (2022). Physiological Profiling and Energy System Contributions During Simulated Epée Matches in Elite Fencers. International Journal of Sports Physiology and Performance, 17(6), 943-950 |
| 1. Flatt, A. A., & Howells, D. (2022). Effects of Long-Haul Travel and the Olympic Games on Heart-Rate Variability in Rugby Sevens Medalists. International Journal of Sports Physiology and Performance, 17(6), 951-960 |
| 1. Henderson, M. J., Chrismas, B. C., Fransen, J., Coutts, A. J., & Taylor, L. (2022). Responses to a 5-Day Sport-Specific Heat Acclimatization Camp in Elite Female Rugby Sevens Athletes. International Journal of Sports Physiology and Performance, 17(6), 969-978. |
| 1. Alabdulwahed, S., Galán-López, N., Hill, T., James, L. J., Chrismas, B. C. R., Racinais, S., Stellingwerff, T., Leal, D. V., Hausen, M., Chamari, K., Fullagar, H. H., Esh, C., & Taylor, L. (2022). Heat Adaptation and Nutrition Practices: Athlete and Practitioner Knowledge and Use. International Journal of Sports Physiology and Performance, 17(7), 1011-1024. |
| 1. Valenzuela, P. L., Mateo-March, M., Zabala, M., Muriel, X., Lucia, A., Barranco-Gil, D., & Pallarés, J. G. (2022). Ambient Temperature and Field-Based Cycling Performance: Insights From Male and Female Professional Cyclists. International Journal of Sports Physiology and Performance, 17(7), 1025-1029 |
| 1. Lipski, E. S., Spindler, D. J., Hesselink, M. K., Myers, T. D., & Sanders, D. (2022). Differences in Performance Assessments Conducted Indoors and Outdoors in Professional Cyclists. International Journal of Sports Physiology and Performance, 17(7), 1054-1060. |
| 1. Lum, D., Soh, S. K., Teo, C. J., Wong, O. Q., & Lee, M. J. (2022). Effects of Performing Isometric Bench Press Training at Single Versus Multiple Joint Positions on Strength and Power Performance. International Journal of Sports Physiology and Performance, 17(7), 1061-1069. |
| 1. van Erp, T., & Lamberts, R. P. (2022). Performance Characteristics of TOP5 Versus NOT-TOP5 Races in Female Professional Cycling. International Journal of Sports Physiology and Performance, 17(7), 1070-1076. |
| 1. Gallo, G., Geda, E., Codella, R., Faelli, E., Panascì, M., Ranieri, L. E., Pollastri, L., Brighenti, S., Molino, L., Riba, U., Luzi, L., Ruggeri, P., & Filipas, L. (2022). Effects of Bilateral Dorsolateral Prefrontal Cortex High-Definition Transcranial Direct-Current Stimulation on Physiological and Performance Responses at Severe-Intensity Exercise Domain in Elite Road Cyclists. International Journal of Sports Physiology and Performance, 17(7), 1085-1093 |
| 1. Gallo, G., Mateo-March, M., Leo, P., Campos-Donaire, A., Gandia-Soriano, A., Giorgi, A., Faelli, E., Ruggeri, P., Codella, R., Mujika, I., & Filipas, L. (2022). Power Road-Derived Physical Performance Parameters in Junior, Under-23, and Professional Road Cycling Climbers. International Journal of Sports Physiology and Performance, 17(7), 1094-1102 |
| 1. Lindberg, K., Solberg, P., Bjørnsen, T., Helland, C., Rønnestad, B., Thorsen Frank, M., Haugen, T., Østerås, S., Kristoffersen, M., Midttun, M., Sæland, F., Eythorsdottir, I., & Paulsen, G. (2022). Strength and Power Testing of Athletes: A Multicenter Study of Test–Retest Reliability. International Journal of Sports Physiology and Performance, 17(7), 1103-1110. |
| 1. Bellinger, P., Lievens, E., Kennedy, B., Rice, H., Derave, W., & Minahan, C. (2022). The Muscle Typology of Elite and World-Class Swimmers. International Journal of Sports Physiology and Performance, 17(8), 1179-1186 |
| 1. García-Fresneda, A., Carmona, G., Yanci, J., & Iturricastillo, A. (2022). Initial Maximum Push-Rim Propulsion and Sprint Performance in Elite Women’s Wheelchair Basketball: Differences Between Players’ Functional Classification. International Journal of Sports Physiology and Performance, 17(8), 1187-1195 |
| 1. Philippe, K., Paillard, T., Maurelli, O., Moody, J., & Prioux, J. (2022). Effects of an Offshore Sailing Competition on Anthropometry, Muscular Performance, Subjective Wellness, and Salivary Cortisol in Professional Sailors. International Journal of Sports Physiology and Performance, 17(8), 1205-1212 |
| 1. Nicol, E., Pearson, S., Saxby, D., Minahan, C., & Tor, E. (2022). The Association of Range of Motion, Dryland Strength–Power, Anthropometry, and Velocity in Elite Breaststroke Swimmers. International Journal of Sports Physiology and Performance, 17(8), 1222-1230. |
| 1. Washif, J. A., Sandbakk, Ø., Seiler, S., Haugen, T., Farooq, A., Quarrie, K., Janse van Rensburg, D. C., Krug, I., Verhagen, E., Wong, D. P., Mujika, I., Cortis, C., Haddad, M., Ahmadian, O., Al Jufaili, M., Al-Horani, R. A., Al-Mohannadi, A. S., Aloui, A., Ammar, A., Arifi, F., Aziz, A. R., Batuev, M., Beaven, C. M., Beneke, R., Bici, A., Bishnoi, P., Bogwasi, L., Bok, D., Boukhris, O., Boullosa, D., Bragazzi, N., Brito, J., Palacios Cartagena, R. P., Chaouachi, A., Cheung, S. S., Chtourou, H., Cosma, G., Debevec, T., DeLang, M. D., Dellal, A., Dönmez, G., Driss, T., Peña Duque, J. D., Eirale, C., Elloumi, M., Foster, C., Franchini, E., Fusco, A., Galy, O., Gastin, P. B., Gill, N., Girard, O., Gregov, C., Halson, S., Hammouda, O., Hanzlíková, I., Hassanmirzaei, B., Hébert-Losier, K., Muñoz Helú, H., Herrera-Valenzuela, T., Hettinga, F. J., Holtzhausen, L., Hue, O., Dello Iacono, A., Ihalainen, J. K., James, C., Joseph, S., Kamoun, K., Khaled, M., Khalladi, K., Kim, K. J., Kok, L., MacMillan, L., Mataruna-Dos-Santos, L. J., Matsunaga, R., Memishi, S., Millet, G. P., Moussa-Chamari, I., Musa, D. I., Nguyen, H. M. T., Nikolaidis, P. T., Owen, A., Padulo, J., Pagaduan, J. C., Perera, N. P., Pérez-Gómez, J., Pillay, L., Popa, A., Pudasaini, A., Rabbani, A., Rahayu, T., Romdhani, M., Salamh, P., Sarkar, A., Schillinger, A., Setyawati, H., Shrestha, N., Suraya, F., Tabben, M., Trabelsi, K., Urhausen, A., Valtonen, M., Weber, J., Whiteley, R., Zrane, A., Zerguini, Y., Zmijewski, P., Ben Saad, H., Pyne, D. B., Taylor, L., & Chamari, K. (2022). COVID-19 Lockdown: A Global Study Investigating the Effect of Athletes’ Sport Classification and Sex on Training Practices. International Journal of Sports Physiology and Performance, 17(8), 1242-1256 |
| 1. Escobar-Álvarez, J. A., Jiménez-Reyes, P., Da Conceição, F. A., & Fuentes-García, J. P. (2022). Effect of Supplementary Physical Training on Vertical Jump Height in Professional Ballet Dancers. International Journal of Sports Physiology and Performance, 17(8), 1257-1263. |
| 1. Lindberg, K., Solberg, P., Bjørnsen, T., Helland, C., Rønnestad, B., Thorsen Frank, M., Haugen, T., Østerås, S., Kristoffersen, M., Midttun, M., Sæland, F., Eythorsdottir, I., & Paulsen, G. (2022). Strength and Power Testing of Athletes: Associations of Common Assessments Over Time. International Journal of Sports Physiology and Performance, 17(8), 1280-1288 |
| 1. Sollie, O., & Losnegard, T. (2022). Sex Differences in Physiological Determinants of Performance in Elite Adolescent, Junior, and Senior Cross-Country Skiers. International Journal of Sports Physiology and Performance, 17(8), 1304-1311 |
| 1. Sanders, D., Spindler, D. J., & Stanley, J. (2022). The Multidisciplinary Physical Preparation of a Multiple Paralympic Medal-Winning Cyclist. International Journal of Sports Physiology and Performance, 17(8), 1316-1322. |
| 1. Biggins, M., Purtill, H., Fowler, P., O’Sullivan, K., & Cahalan, R. (2022). Impact of Long-Haul Travel to International Competition on Sleep and Recovery in Elite Male and Female Soccer Athletes. International Journal of Sports Physiology and Performance, 17(9), 1361-1370. |
| 1. Salhi, I., Ben Aabderrahman, A., Triki, R., Clark, C. C., Gaed, S., Hackney, A. C., Saeidi, A., Laher, I., Kurtz, J. A., VanDusseldorp, T. A., & Zouhal, H. (2022). Gastrointestinal Hormones, Morphological Characteristics, and Physical Performance in Elite Soccer Players. International Journal of Sports Physiology and Performance, 17(9), 1371-1381. |
| 1. Angius, L., Merlini, M., Hopker, J., Bianchi, M., Fois, F., Piras, F., Cugia, P., Russell, J., & Marcora, S. M. (2022). Physical and Mental Fatigue Reduce Psychomotor Vigilance in Professional Football Players. International Journal of Sports Physiology and Performance, 17(9), 1391-1398 |
| 1. Delaval, B., Abaïdia, A., Delecroix, B., Le Gall, F., McCall, A., Ahmaidi, S., & Dupont, G. (2022). Recovery During a Congested Schedule and Injury in Professional Football. International Journal of Sports Physiology and Performance, 17(9), 1399-1406 |
| 1. Light, N., Thorborg, K., Krommes, K., Nielsen, M. F., Thornton, K. B., Hölmich, P., Penalver, J. J., & Ishøi, L. (2022). Rapid Spike in Hip Adduction Strength in Early Adolescent Footballers: A Study of 125 Elite Male Players From Youth to Senior. International Journal of Sports Physiology and Performance, 17(9), 1407-1414. |
| 1. Houtmeyers, K. C., Robberechts, P., Jaspers, A., McLaren, S. J., Brink, M. S., Vanrenterghem, J., Davis, J. J., & Helsen, W. F. (2022). Differential Ratings of Perceived Exertion: Relationships With External Intensity and Load in Elite Men’s Football. International Journal of Sports Physiology and Performance, 17(9), 1415-1424. |
| 1. Segers, N., Waldron, M., Howe, L. P., Patterson, S. D., Moran, J., Jones, B., Kidgell, D. J., & Tallent, J. (2022). Slow-Speed Compared With Fast-Speed Eccentric Muscle Actions Are Detrimental to Jump Performance in Elite Soccer Players In-Season. International Journal of Sports Physiology and Performance, 17(9), 1425-1431 |
| 1. Ruiz-Navarro, J. J., Gay, A., Zacca, R., Cuenca-Fernández, F., López-Belmonte, Ó., López-Contreras, G., Morales-Ortiz, E., & Arellano, R. (2022). Biophysical Impact of 5-Week Training Cessation on Sprint Swimming Performance. International Journal of Sports Physiology and Performance, 17(10), 1463-1472. |
| 1. García, F., Fernández, D., & Martín, L. (2022). Relationship Between Game Load and Player’s Performance in Professional Basketball. International Journal of Sports Physiology and Performance, 17(10), 1473-1479 |
| 1. Bardin, J., Bourdier, P., Bontemps, B., Diry, A., Birat, A., Blazevich, A. J., Roualen, F., Hanon, C., Thomas, C., & Ratel, S. (2022). Repeated Simulated Match-Induced Changes in Finger Flexor Force and Blood Acid–Base Balance in World-Class Female Judokas. International Journal of Sports Physiology and Performance, 17(10), 1499-1506 |
| 1. Sommer Jeppesen, J., Vigh-Larsen, J. F., Oxfeldt, M. S., Laustsen, N. M., Mohr, M., Bangsbo, J., & Hostrup, M. (2022). Four Weeks of Intensified Training Enhances On-Ice Intermittent Exercise Performance and Increases Maximal Oxygen Consumption of Youth National-Team Ice Hockey Players. International Journal of Sports Physiology and Performance, 17(10), 1507-1515 |
| 1. Li, Q., Steward, C. J., Cullen, T., Che, K., & Zhou, Y. (2022). Presleep Heart-Rate Variability Biofeedback Improves Mood and Sleep Quality in Chinese Winter Olympic Bobsleigh Athletes. International Journal of Sports Physiology and Performance, 17(10), 1516-1526 |
| 1. Suppiah, H. T., Gastin, P. B., & Driller, M. W. (2022). A Strategy to Inform Athlete Sleep Support From Questionnaire Data and Its Application in an Elite Athlete Cohort. International Journal of Sports Physiology and Performance, 17(10), 1532-1536 |
| 1. Garzon, M., Leguizamo, J., Saldarriaga, F., Galeano, E., & Millet, G. P. (2022). Does Altitude of Birth Influence the Performance of National- to Elite-Level Colombian Cyclists?. International Journal of Sports Physiology and Performance, 17(12), 1756-1759 |
| 1. Stephenson, B. T., O’Brien, T. J., Hutchinson, M. J., D’Angeli, C., Cockram, A., Mason, B. S., & Goosey-Tolfrey, V. L. (2022). Ice Slurry Ingestion Lowers Thermoregulatory Strain in Wheelchair Tennis Players During Repeated Sprint Intervals in the Heat. International Journal of Sports Physiology and Performance, 17(12), 1748-1755 |
| 1. Boone, J., Caen, K., Lievens, M., Bourgois, G., Colosio, A. L., & Bourgois, J. G. (2022). Physical Preparation of a World-Class Lightweight Men’s Double Sculls Team for the Tokyo 2020 Olympics. International Journal of Sports Physiology and Performance, 17(12), 1741-1747. |
| 1. Staiano, W., Merlini, M., Romagnoli, M., Kirk, U., Ring, C., & Marcora, S. (2022). Brain Endurance Training Improves Physical, Cognitive, and Multitasking Performance in Professional Football Players. International Journal of Sports Physiology and Performance, 17(12), 1732-1740. |
| 1. Goodrich, J. A., Frisco, D. J., Kim, S., VanBaak, K., Holliday, M., Rueda, M., Poddar, S., & Byrnes, W. C. (2022). Iron Status and Homeostasis Across 2 Competitive Seasons in NCAA Division I Collegiate Cross-Country Runners Residing at Low Altitude. International Journal of Sports Physiology and Performance, 17(12), 1716-1724 |
| 1. Majumder, T., De Martin Topranin, V., Sandbakk, Ø., & Noordhof, D. A. (2022). Indian Endurance Athletes’ Menstrual Cycle: Practices, Knowledge, Communication, Health, and Changes in Perceptions Across the Phases. International Journal of Sports Physiology and Performance, 17(12), 1706-1715 |
| 1. Legg, K. A., Cochrane, D. J., Gee, E. K., Macdermid, P. W., & Rogers, C. W. (2022). Physiological Demands and Muscle Activity of “Track-Work” Riding in Apprentice Jockeys. International Journal of Sports Physiology and Performance, 17(12), 1698-1705 |
| 1. Letter, R. T., Dwyer, D. B., Drinkwater, E. J., & Feros, S. A. (2022). The Physical Qualities of Elite Australian Pace Bowlers: Typical Characteristics and Longitudinal Changes in Men and Women. International Journal of Sports Physiology and Performance, 17(12), 1691-1697 |
| 1. Henze, A., Huth, J., & Mauch, F. (2022). Biochemical Monitoring of Muscle Recovery in Elite Handball Using an Individualized Approach. International Journal of Sports Physiology and Performance, 17(12), 1683-1690 |
| 1. Seeberg, T. M., Kocbach, J., Kjøsen Talsnes, R., Meyer, F., Losnegard, T., Tjønnås, J., Sandbakk, Ø., & Solli, G. S. (2022). Performance Effects of Video- and Sensor-Based Feedback for Implementing a Terrain-Specific Micropacing Strategy in Cross-Country Skiing. International Journal of Sports Physiology and Performance, 17(12), 1672-1682. |
| 1. Staunton, C. A., Andersson, E. P., Skovereng, K., & Björklund, G. (2022). Heart Rate Does Not Accurately Predict Metabolic Intensity During Variable-Intensity Roller Skiing or Cycling. International Journal of Sports Physiology and Performance, 17(12), 1664-1671 |
| 1. Finlay, M. J., Bridge, C. A., Greig, M., & Page, R. M. (2022). Postactivation Performance Enhancement of Amateur Boxers’ Punch Force and Neuromuscular Performance Following 2 Upper-Body Conditioning Activities. International Journal of Sports Physiology and Performance, 17(11), 1621-1633 |
| 1. Fessl, I., Dirnberger, J., Kröll, J., & Wiesinger, H. (2022). Isokinetic Leg-Press Power–Force–Velocity Profiles Are Reliable in Male and Female Elite Athletes but Not Interchangeable With Vertical Jump Profiles. International Journal of Sports Physiology and Performance, 17(11), 1614-1620 |
| 1. Pugh, C. F., Beaven, C. M., Ferguson, R. A., Driller, M. W., Palmer, C. D., & Paton, C. D. (2022). Critical Power, Work Capacity, and Recovery Characteristics of Team-Pursuit Cyclists. International Journal of Sports Physiology and Performance, 17(11), 1606-1613 |
| 1. Simpson, M. J., Jenkins, D. G., Connick, M., & Kelly, V. G. (2022). Relationship Between Training Workloads, Match Workloads, and Match Performance in Elite Netball. International Journal of Sports Physiology and Performance, 17(11), 1599-1605 |
| 1. Talsnes, R. K., Nordgården, S., Kocbach, J., & Solli, G. S. (2022). One Long Versus 2 Short Sessions? Physiological and Perceptual Responses to Low-Intensity Training at Self-Selected Speeds in Cross-Country Skiers. International Journal of Sports Physiology and Performance, 17(11), 1590-1598 |
| 1. Duc, S., Urianstad, T., & Rønnestad, B. R. (2022). Adding Vibration During Varied-Intensity Work Intervals Increases Time Spent Near Maximal Oxygen Uptake in Well-Trained Cyclists. International Journal of Sports Physiology and Performance, 17(11), 1565-1573 |
| 1. Pallares, J. G., Hernández-Belmonte, A., Valenzuela, P. L., Muriel, X., Mateo-March, M., Barranco-Gil, D., & Lucia, A. (2022). Field-Derived Maximal Power Output in Cycling: An Accurate Indicator of Maximal Performance Capacity?. International Journal of Sports Physiology and Performance, 17(11), 1558-1564 |
| 1. Shibata, S., Kageyama, M., Inaba, Y., Yoshioka, S., & Fukashiro, S. (2022). Kinetic analysis of the wrist and fingers during fastball and curveball pitches. European journal of sport science, 22(2), 136–145. https://doi.org/10.1080/17461391.2020.1866080 |
| 1. Lopes-Silva, J. P., Rocha, A. L. S. D., Rocha, J. C. C., Silva, V. F. D. S., & Correia-Oliveira, C. R. (2022). Caffeine ingestion increases the upper-body intermittent dynamic strength endurance performance of combat sports athletes. European journal of sport science, 22(2), 227–236. https://doi.org/10.1080/17461391.2021.1874058 |
| 1. Pété, E., Leprince, C., Lienhart, N., & Doron, J. (2022). Dealing with the impact of the COVID-19 outbreak: Are some athletes' coping profiles more adaptive than others?. European journal of sport science, 22(2), 237–247. https://doi.org/10.1080/17461391.2021.1873422 |
| 1. Hopkinson, M., Nicholson, G., Weaving, D., Hendricks, S., Fitzpatrick, A., Naylor, A., Robertson, C., Beggs, C., & Jones, B. (2022). Rugby league ball carrier injuries: The relative importance of tackle characteristics during the European Super League. European journal of sport science, 22(2), 269–278. https://doi.org/10.1080/17461391.2020.1853817 |
| 1. Bliss, A., Waldron, M., & Maxwell, N. (2022). Predicting middle-distance track and cross-country performances of national and international level adolescent runners. European journal of sport science, 22(3), 305–313. https://doi.org/10.1080/17461391.2020.1867650 |
| 1. Birdsey, L. P., Weston, M., Russell, M., Johnston, M., Cook, C. J., & Kilduff, L. P. (2022). The neuromuscular, physiological, endocrine and perceptual responses to different training session orders in international female netball players. European journal of sport science, 22(3), 314–325. https://doi.org/10.1080/17461391.2020.1869837 |
| 1. Naito, T., Nakamura, M., Muraishi, K., Eda, N., Ando, K., Takemura, A., Akazawa, N., Hasegawa, H., & Takahashi, H. (2022). In-play optimal cooling for outdoor match-play tennis in the heat. European journal of sport science, 22(3), 326–335. https://doi.org/10.1080/17461391.2020.1870160 |
| 1. Mon-López, D., Bernardez-Vilaboa, R., Sillero-Quintana, M., & Alvarez Fernandez-Balbuena, A. (2022). Air shooting competition effects on visual skills depending on the sport level. European journal of sport science, 22(3), 336–343. https://doi.org/10.1080/17461391.2021.1874540 |
| 1. Stojanović, E., Scanlan, A. T., Milanović, Z., Fox, J. L., Stanković, R., & Dalbo, V. J. (2022). Acute caffeine supplementation improves jumping, sprinting, and change-of-direction performance in basketball players when ingested in the morning but not evening. European journal of sport science, 22(3), 360–370. https://doi.org/10.1080/17461391.2021.1874059 |
| 1. Possamai, L. T., Borszcz, F. K., de Aguiar, R. A., de Lucas, R. D., & Turnes, T. (2022). Agreement of maximal lactate steady state with critical power and physiological thresholds in rowing. European journal of sport science, 22(3), 371–380. https://doi.org/10.1080/17461391.2021.1874541 |
| 1. Baldassarre, R., Ieno, C., Bonifazi, M., Di Castro, A., Gianfelici, A., & Piacentini, M. F. (2022). Carbohydrate supplementation during a simulated 10-km open water swimming race: effects on physiological, perceptual parameters and performance. European journal of sport science, 22(3), 390–398. https://doi.org/10.1080/17461391.2021.1880644 |
| 1. Chen, Y. T., Hsieh, Y. Y., Ho, J. Y., Lin, T. Y., & Lin, J. C. (2022). Two weeks of detraining reduces cardiopulmonary function and muscular fitness in endurance athletes. European journal of sport science, 22(3), 399–406. https://doi.org/10.1080/17461391.2021.1880647 |
| 1. Júdice, P. B., Hetherington-Rauth, M., Magalhães, J. P., Correia, I. R., & Sardinha, L. B. (2022). Sedentary behaviours and their relationship with body composition of athletes. European journal of sport science, 22(3), 474–480. https://doi.org/10.1080/17461391.2021.1874060 |
| 1. de Leeuw, A. W., van der Zwaard, S., van Baar, R., & Knobbe, A. (2022). Personalized machine learning approach to injury monitoring in elite volleyball players. European journal of sport science, 22(4), 511–520. https://doi.org/10.1080/17461391.2021.1887369 |
| 1. Brown, F. C. W., Hill, J. A., van Someren, K., Howatson, G., & Pedlar, C. R. (2022). The effect of custom-fitted compression garments worn overnight for recovery from judo training in elite athletes. European journal of sport science, 22(4), 521–529. https://doi.org/10.1080/17461391.2021.1891294 |
| 1. Hogan, C., Binnie, M. J., Doyle, M., & Peeling, P. (2022). Mean maximal power from an on-water 1000-m time-trial predicts lactate threshold power in well-trained flat-water sprint kayak athletes. European journal of sport science, 22(4), 549–558. https://doi.org/10.1080/17461391.2021.1880648 |
| 1. Karuk, H. N., Rudarli Nalcakan, G., & Pekünlü, E. (2022). Effects of carbohydrate and caffeine combination mouth rinse on anaerobic performance of highly trained male athletes. European journal of sport science, 22(4), 589–599. https://doi.org/10.1080/17461391.2021.1907449 |
| 1. Klatt, S., & Smeeton, N. J. (2022). Processing visual information in elite junior soccer players: Effects of chronological age and training experience on visual perception, attention, and decision making. European journal of sport science, 22(4), 600–609. https://doi.org/10.1080/17461391.2021.1887366 |
| 1. Kordi, M., Galis, G., Erp, T. V., & Terra, W. (2022). Reliability and sensitivity of the Notio Konect to quantify coefficient of drag area in elite track cyclists. European journal of sport science, 22(6), 774–779. https://doi.org/10.1080/17461391.2021.1891296 |
| 1. Okumura, F., Yokoyama, K., & Yamamoto, Y. (2022). State transitions among groups of cyclists in cycling points races. European journal of sport science, 22(6), 790–798. https://doi.org/10.1080/17461391.2021.1905077 |
| 1. Takahashi, K., Kamibayashi, K., & Wakahara, T. (2022). Gluteus and posterior thigh muscle sizes in sprinters: Their distributions along muscle length. European journal of sport science, 22(6), 799–807. https://doi.org/10.1080/17461391.2021.1907450 |
| 1. Bertozzi, F., Porcelli, S., Marzorati, M., Pilotto, A. M., Galli, M., Sforza, C., & Zago, M. (2022). Whole-body kinematics during a simulated sprint in flat-water kayakers. European journal of sport science, 22(6), 817–825. https://doi.org/10.1080/17461391.2021.1930190 |
| 1. Zimmermann, H. B., Costa, F. E., Sakugawa, R., MacIntosh, B., Diefenthaeler, F., & Dal Pupo, J. (2022). Plyometric exercise enhances twitch contractile properties but fails to improve voluntary rate of torque development in highly trained sprint athletes. European journal of sport science, 22(6), 857–866. https://doi.org/10.1080/17461391.2021.1916083 |
| 1. Russell, S., Jenkins, D. G., Halson, S. L., Juliff, L. E., & Kelly, V. G. (2022). How do elite female team sport athletes experience mental fatigue? Comparison between international competition, training and preparation camps. European journal of sport science, 22(6), 877–887. https://doi.org/10.1080/17461391.2021.1897165 |
| 1. Rumbold, J. L., Newman, J. A., Foster, D., Rhind, D. J. A., Phoenix, J., & Hickey, L. (2022). Assessing post-game emotions in soccer teams: The role of distinct emotional dynamics. European journal of sport science, 22(6), 888–896. https://doi.org/10.1080/17461391.2021.1916079 |
| 1. Ballesio, A., Vacca, M., Bacaro, V., Benazzi, A., De Bartolo, P., Alivernini, F., Lucidi, F., Lombardo, C., & Baglioni, C. (2022). Psychological correlates of insomnia in professional soccer players: An exploratory study. European journal of sport science, 22(6), 897–905. https://doi.org/10.1080/17461391.2021.1892197 |
| 1. Dunn, E. C., Humberstone, C. E., Franchini, E., Iredale, F. K., & Blazevich, A. J. (2022). The effect of fatiguing lower-body exercise on punch forces in highly-trained boxers. European journal of sport science, 22(7), 964–972. https://doi.org/10.1080/17461391.2021.1916085 |
| 1. Wiewelhove, T., Szwajca, S., Busch, M., Döweling, A., Volk, N. R., Schneider, C., Meyer, T., Kellmann, M., Pfeiffer, M., & Ferrauti, A. (2022). Recovery during and after a simulated multi-day tennis tournament: Combining active recovery, stretching, cold-water immersion, and massage interventions. European journal of sport science, 22(7), 973–984. https://doi.org/10.1080/17461391.2021.1936196 |
| 1. Olsson, L. F., Madigan, D. J., Hill, A. P., & Grugan, M. C. (2022). Do Athlete and Coach Performance Perfectionism Predict Athlete Burnout?. European journal of sport science, 22(7), 1073–1084. https://doi.org/10.1080/17461391.2021.1916080 |
| 1. Ramírez-López, C., Till, K., Weaving, D., Boyd, A., Peeters, A., Beasley, G., Bradley, S., Giuliano, P., Venables, C., & Jones, B. (2022). Does perceived wellness influence technical-tactical match performance? A study in youth international rugby using partial least squares correlation analysis. European journal of sport science, 22(7), 1085–1093. https://doi.org/10.1080/17461391.2021.1936195 |
| 1. Freitas, S. R., Mendes, B., Firmino, T., Correia, J. P., Witvrouw, E. E. M. C., Oliveira, R., & Vaz, J. R. (2022). Semitendinosus and biceps femoris long head active stiffness response until failure in professional footballers with vs. without previous hamstring injury. European journal of sport science, 22(7), 1132–1140. https://doi.org/10.1080/17461391.2021.1910347 |
| 1. Hermosilla, F., Yustres, I., Psycharakis, S., Santos Del Cerro, J., González-Mohíno, F., & González-Rave, J. M. (2022). Which variables may affect underwater glide performance after a swimming start?. European journal of sport science, 22(8), 1141–1148. https://doi.org/10.1080/17461391.2021.1944322 |
| 1. Etxebarria, N., Spratford, W., Iriberri, J., Ross, M., Gomez-Ezeiza, J., & Pyne, D. (2022). Energetics in elite race walkers. European journal of sport science, 22(8), 1149–1155. https://doi.org/10.1080/17461391.2021.1948615 |
| 1. Springham, M., Williams, S., Waldron, M., McLellan, C., & Newton, R. U. (2022). Summated training and match load predictors of salivary immunoglobulin-A, alpha-amylase, testosterone, cortisol and T:C profile changes in elite-level professional football players: A longitudinal analysis. European journal of sport science, 22(8), 1156–1166. https://doi.org/10.1080/17461391.2021.1949638 |
| 1. Dolci, F., Kilding, A., Spiteri, T., Chivers, P., Piggott, B., Maiorana, A., & Hart, N. H. (2022). Characterising running economy and change of direction economy between soccer players of different playing positions, levels and sex. European journal of sport science, 22(8), 1167–1176. https://doi.org/10.1080/17461391.2021.1953151 |
| 1. Craven, J., Cox, A. J., Bellinger, P., Desbrow, B., Irwin, C., Buchan, J., McCartney, D., & Sabapathy, S. (2022). The influence of exercise training volume alterations on the gut microbiome in highly-trained middle-distance runners. European journal of sport science, 22(8), 1222–1230. https://doi.org/10.1080/17461391.2021.1933199 |
| 1. Inoue, A., do Carmo, E. C., de Souza Terra, B., Moraes, B. R., Lattari, E., & Borin, J. P. (2022). Comparison of coach-athlete perceptions on internal and external training loads in trained cyclists. European journal of sport science, 22(8), 1261–1267. https://doi.org/10.1080/17461391.2021.1927198 |
| 1. Teune, B., Woods, C., Sweeting, A., Inness, M., & Robertson, S. (2022). The influence of environmental and task constraint interaction on skilled behaviour in Australian Football. European journal of sport science, 22(8), 1268–1275. https://doi.org/10.1080/17461391.2021.1958011 |
| 1. Vaamonde, D., García-Manso, J. M., Algar-Santacruz, C., Abbasi, A., Sarmiento, S., & Valverde-Esteve, T. (2022). Behaviour of salivary testosterone and cortisol in men during an Ironman Triathlon. European journal of sport science, 22(9), 1335–1342. https://doi.org/10.1080/17461391.2021.1955011 |
| 1. D'Hondt, J., Chapelle, L., Droogenbroeck, L. V., Aerenhouts, D., Clarys, P., & D'Hondt, E. (2022). Bioelectrical impedance analysis as a means of quantifying upper and lower limb asymmetry in youth elite tennis players: An explorative study. European journal of sport science, 22(9), 1343–1354. https://doi.org/10.1080/17461391.2021.1960624 |
| 1. Zignoli, A., Biral, F., Fornasiero, A., Sanders, D., Erp, T. V., Mateo-March, M., Fontana, F. Y., Artuso, P., Menaspà, P., Quod, M., Giorgi, A., & Laursen, P. B. (2022). Assessment of bike handling during cycling individual time trials with a novel analytical technique adapted from motorcycle racing. European journal of sport science, 22(9), 1355–1363. https://doi.org/10.1080/17461391.2021.1966517 |
| 1. Brink, N. J., Constantinou, D., & Torres, G. (2022). Postactivation performance enhancement (PAPE) of sprint acceleration performance. European journal of sport science, 22(9), 1411–1417. https://doi.org/10.1080/17461391.2021.1955012 |
| 1. Tsai, C. L., Ju, J., & Chen, Z. (2022). The mediating role of prosocial and antisocial behaviors between team trust and sport commitment in college basketball players. European journal of sport science, 22(9), 1418–1425. https://doi.org/10.1080/17461391.2021.1973571 |
| 1. Sharps, F. R. J., Wilson, L. J., Graham, C. A., & Curtis, C. (2022). Prevalence of disordered eating, eating disorders and risk of low energy availability in professional, competitive and recreational female athletes based in the United Kingdom. European journal of sport science, 22(9), 1445–1451. https://doi.org/10.1080/17461391.2021.1943712 |
| 1. Pastor, T., Schweizer, A., Reissner, L., Pastor, T., Spörri, J., & Fröhlich, S. (2022). Long-term evolution of cartilage abnormalities and osteophytes in the fingers of elite sport climbers: A cross-sectional 10-year follow-up study. European journal of sport science, 22(9), 1452–1458. https://doi.org/10.1080/17461391.2021.1943716 |
| 1. Morrison, B., Mohammad, A., Oxborough, D., Somauroo, J., Lindsay, S., Drane, A. L., Shave, R., & George, K. (2022). The 12-lead electrocardiogram of the elite female footballer as defined by different interpretation criteria across the competitive season. European journal of sport science, 22(10), 1475–1483. https://doi.org/10.1080/17461391.2021.1966103 |
| 1. Lang, D., & Zhou, A. (2022). Relationships between postural balance, aiming technique and performance in elite rifle shooters. European journal of sport science, 22(10), 1493–1498. https://doi.org/10.1080/17461391.2021.1971775 |
| 1. Fenemor, S. P., Gill, N. D., Driller, M. W., Mills, B., Casadio, J. R., & Beaven, C. M. (2022). The relationship between physiological and performance variables during a hot/humid international rugby sevens tournament. European journal of sport science, 22(10), 1499–1507. https://doi.org/10.1080/17461391.2021.1973111 |
| 1. Stojanović, E., Jakovljević, V., Scanlan, A. T., Dalbo, V. J., & Radovanović, D. (2022). Vitamin D3 supplementation reduces serum markers of bone resorption and muscle damage in female basketball players with vitamin D inadequacy. European journal of sport science, 22(10), 1532–1542. https://doi.org/10.1080/17461391.2021.1953153 |
| 1. Voet, J. G., Lamberts, R. P., de Koning, J. J., de Jong, J., Foster, C., & van Erp, T. (2022). Differences in execution and perception of training sessions as experienced by (semi-) professional cyclists and their coach. European journal of sport science, 22(10), 1586–1594. https://doi.org/10.1080/17461391.2021.1979102 |
| 1. Inoue, A., Lattari, E., do Carmo, E. C., Rodrigues, G. M., de Oliveira, B. R. R., & Santos, T. M. (2022). Correlation between economy/efficiency and mountain biking cross-country race performance. European journal of sport science, 22(11), 1641–1648. https://doi.org/10.1080/17461391.2021.1968504 |
| 1. Dutton, M., Gray, J., Divekar, N., Prins, D., & Tam, N. (2022). Overhead throwing biomechanics in cricketers: The effect of a run-up approach. European journal of sport science, 22(11), 1686–1694. https://doi.org/10.1080/17461391.2021.1979103 |
| 1. Hurst, P., Ring, C., & Kavussanu, M. (2022). Ego orientation is related to doping likelihood via sport supplement use and sport supplement beliefs. European journal of sport science, 22(11), 1734–1742. https://doi.org/10.1080/17461391.2021.1995509 |
| 1. Muriel, X., Mateo-March, M., Valenzuela, P. L., Zabala, M., Lucia, A., Pallares, J. G., & Barranco-Gil, D. (2022). Durability and repeatability of professional cyclists during a Grand Tour. European journal of sport science, 22(12), 1797–1804. https://doi.org/10.1080/17461391.2021.1987528 |
| 1. Gómez-Carmona, C. D., Mancha-Triguero, D., Pino-Ortega, J., & Ibáñez, S. J. (2022). Characterization and sex-related differences in the multi-location external workload profile of semiprofessional basketball players. A cross-sectional study. European journal of sport science, 22(12), 1816–1826. https://doi.org/10.1080/17461391.2021.2009040 |
| 1. Keaney, L. C., Kilding, A. E., Merien, F., Shaw, D. M., & Dulson, D. K. (2022). Upper respiratory tract symptom risk in elite field hockey players during a dry run for the Tokyo Olympics. European journal of sport science, 22(12), 1827–1835. https://doi.org/10.1080/17461391.2021.2009041 |
| 1. Grimson, S., Brickley, G., Smeeton, N. J., Abbott, W., & Brett, A. (2022). Physical activity on mental wellbeing in senior English Premier League soccer players during the COVID-19 pandemic and the lockdown. European journal of sport science, 22(12), 1916–1925. https://doi.org/10.1080/17461391.2021.1976841 |
| 1. Navia, J. A., Avilés, C., Dicks, M., & Ruiz-Pérez, L. M. (2022). The spatiotemporal control of expert tennis players when returning first serves: A perception-action perspective. Journal of sports sciences, 40(1), 16–23. https://doi.org/10.1080/02640414.2021.1976484 |
| 1. Cook, G. M., Fletcher, D., & Peyrebrune, M. (2022). Olympic coaching excellence: A quantitative study of Olympic swimmers' perceptions of their coaches. Journal of sports sciences, 40(1), 32–39. https://doi.org/10.1080/02640414.2021.1976486 |
| 1. Atack, A. C., Trewartha, G., & Bezodis, N. E. (2022). The approach towards the ball, rather than the physical characteristics of the kicker, limits accurate rugby place kicking range. Journal of sports sciences, 40(1), 104–115. https://doi.org/10.1080/02640414.2021.1976494 |
| 1. White, R., Palczewska, A., Weaving, D., Collins, N., & Jones, B. (2022). Sequential movement pattern-mining (SMP) in field-based team-sport: A framework for quantifying spatiotemporal data and improve training specificity?. Journal of sports sciences, 40(2), 164–174. https://doi.org/10.1080/02640414.2021.1982484 |
| 1. Wild, J. J., Bezodis, I. N., North, J. S., & Bezodis, N. E. (2022). Characterising initial sprint acceleration strategies using a whole-body kinematics approach. Journal of sports sciences, 40(2), 203–214. https://doi.org/10.1080/02640414.2021.1985759 |
| 1. Krabben, K., Mann, D., Lojanica, M., Mueller, D., Dominici, N., van der Kamp, J., & Savelsbergh, G. (2022). How wide should you view to fight? Establishing the size of the visual field necessary for grip fighting in judo. Journal of sports sciences, 40(2), 236–247. https://doi.org/10.1080/02640414.2021.1987721 |
| 1. Delaney, J. A., McKay, B. A., Radcliffe, J., Benton, D. T., Samozino, P., Morin, J. B., & Duthie, G. M. (2022). Uphill sprinting load- and force-velocity profiling: Assessment and potential applications. Journal of sports sciences, 40(3), 281–287. https://doi.org/10.1080/02640414.2021.1992868 |
| 1. McGrath, J. W., Neville, J., Stewart, T., Clinning, H., Thomas, B., & Cronin, J. (2022). Quantifying cricket fast bowling volume, speed and perceived intensity zone using an Apple Watch and machine learning. Journal of sports sciences, 40(3), 323–330. https://doi.org/10.1080/02640414.2021.1993640 |
| 1. Woodhouse, L. N., Tallent, J., Patterson, S. D., & Waldron, M. (2022). International female rugby union players' anthropometric and physical performance characteristics: A five-year longitudinal analysis by individual positional groups. Journal of sports sciences, 40(4), 370–378. https://doi.org/10.1080/02640414.2021.1993656 |
| 1. Tribolet, R., Sheehan, W. B., Novak, A. R., Rennie, M. J., Watsford, M. L., & Fransen, J. (2022). Match simulation practice may not represent competitive match play in professional Australian football. Journal of sports sciences, 40(4), 413–421. https://doi.org/10.1080/02640414.2021.1995245 |
| 1. Prendergast, G., & Gibson, L. (2022). A qualitative exploration of the use of player loans to supplement the talent development process of professional footballers in the under 23 age group of English football academies. Journal of sports sciences, 40(4), 422–430. https://doi.org/10.1080/02640414.2021.1996985 |
| 1. Runswick, O. R., Mann, D. L., Mand, S., Fletcher, A., & Allen, P. M. (2022). Laterality and performance: Are golfers learning to play backwards?. Journal of sports sciences, 40(4), 450–458. https://doi.org/10.1080/02640414.2021.1997011 |
| 1. St George, L., Thetford, C., Clayton, H. M., & Hobbs, S. J. (2022). An exploration of stakeholder perceptions to inform the development of an evidence-based classification system in para dressage. Journal of sports sciences, 40(4), 459–469. https://doi.org/10.1080/02640414.2021.1997012 |
| 1. Lascu, A., Spratford, W., Pyne, D. B., & Etxebarria, N. (2022). "Train how you play": Using representative learning design to train amateur cricketers. Journal of sports sciences, 40(5), 498–508. https://doi.org/10.1080/02640414.2021.2001160 |
| 1. Doan, B. K., Heaton, K. J., Self, B. P., Butler Samuels, M. A., & Adam, G. E. (2022). Quantifying head impacts and neurocognitive performance in collegiate boxers. Journal of sports sciences, 40(5), 509–517. https://doi.org/10.1080/02640414.2021.2001175 |
| 1. Perrett, C., Bussey, M., & Lamb, P. (2022). External workload intensity in cricket fast bowlers across maximal and submaximal intensities: Modifying PlayerLoad and IMU location. Journal of sports sciences, 40(5), 527–533. https://doi.org/10.1080/02640414.2021.2003570 |
| 1. di Fronso, S., Montesano, C., Costa, S., Santi, G., Robazza, C., & Bertollo, M. (2022). Rebooting in sport training and competitions: Athletes' perceived stress levels and the role of interoceptive awareness. Journal of sports sciences, 40(5), 542–549. https://doi.org/10.1080/02640414.2021.2004679 |
| 1. Isoard-Gautheur, S., Ginoux, C., & Trouilloud, D. (2022). Associations between peer motivational climate and athletes' sport-related well-being: Examining the mediating role of motivation using a multi-level approach. Journal of sports sciences, 40(5), 550–560. https://doi.org/10.1080/02640414.2021.2004680 |
| 1. Poucher, Z. A., Tamminen, K. A., Sabiston, C. M., & Cairney, J. (2022). A longitudinal examination of changes in mental health among elite Canadian athletes. Journal of sports sciences, 40(7), 733–741. https://doi.org/10.1080/02640414.2021.2015908 |
| 1. McBride, C., & Bronner, S. (2022). Injury characteristics in professional modern dancers: A 15-year analysis of work-related injury rates and patterns. Journal of sports sciences, 40(7), 821–837. https://doi.org/10.1080/02640414.2021.2021030 |
| 1. Scott, T. J., McLaren, S. J., Lovell, R., Scott, M. T. U., & Barrett, S. (2022). The reliability, validity and sensitivity of an individualised sub-maximal fitness test in elite rugby league athletes. Journal of sports sciences, 40(8), 840–852. https://doi.org/10.1080/02640414.2021.2021047 |
| 1. Zeff, S., Weir, G., Hamill, J., & van Emmerik, R. (2022). Head control and head-trunk coordination as a function of anticipation in sidestepping. Journal of sports sciences, 40(8), 853–862. https://doi.org/10.1080/02640414.2021.2021683 |
| 1. Virdinli, S. G., Kutlay, E., Yuzbasioglu, Y., Vollaard, N. B. J., & Rudarli Nalcakan, G. (2022). The effect of mouth rinsing with different concentrations of caffeine solutions on reaction time. Journal of sports sciences, 40(8), 928–933. https://doi.org/10.1080/02640414.2022.2038893 |
| 1. Brice, S. M., Millett, E. L., & Philippa, B. (2022). The validity of using inertial measurement units to monitor the torso and pelvis sagittal plane motion of elite rowers. Journal of sports sciences, 40(8), 950–958. https://doi.org/10.1080/02640414.2022.2042146 |
| 1. Duchene, Y., Gauchard, G. C., & Mornieux, G. (2022). Influence of sidestepping expertise and core stability on knee joint loading during change of direction. Journal of sports sciences, 40(9), 959–967. https://doi.org/10.1080/02640414.2022.2042980 |
| 1. Delves, R. I. M., Duthie, G. M., Ball, K. A., & Aughey, R. J. (2022). Applying common filtering processes to Global Navigation Satellite System-derived acceleration during team sport locomotion. Journal of sports sciences, 40(10), 1116–1126. https://doi.org/10.1080/02640414.2022.2051332 |
| 1. Hurst, P., Ring, C., & Kavussanu, M. (2022). Moral values and moral identity moderate the indirect relationship between sport supplement use and doping use via sport supplement beliefs. Journal of sports sciences, 40(10), 1160–1167. https://doi.org/10.1080/02640414.2022.2053387 |
| 1. Padulo, J., Kuvačić, G., Ardigò, L. P., Dhahbi, W., Esposito, F., Samozino, P., & Cè, E. (2022). Bilateral deficit magnitude increases with velocity during a half-squat exercise. Journal of sports sciences, 40(11), 1206–1213. https://doi.org/10.1080/02640414.2022.2051304 |
| 1. Birse, S. M., Webster, K. E., Middleton, K. J., & McClelland, J. A. (2022). Differences in accuracy and consistency in elite lawn bowlers. Journal of sports sciences, 40(11), 1214–1219. https://doi.org/10.1080/02640414.2022.2056311 |
| 1. Eustace, S. J., Morris, R., Tallis, J., Page, R. M., & Greig, M. (2022). The influence of angle-specific torque of the knee flexors and extensors on the angle-specific dynamic control ratio in professional female soccer players. Journal of sports sciences, 40(11), 1235–1242. https://doi.org/10.1080/02640414.2022.2061251 |
| 1. Cartigny, E., Vickers, E., Harrison, G., Appleby, R., & McCullogh, N. (2022). The impact of COVID-19 on dual career athletes: Three typologies of coping. Journal of sports sciences, 40(11), 1265–1274. https://doi.org/10.1080/02640414.2022.2065088 |
| 1. Thomas, R. J., Timmins, R. G., Tofari, P. J., Williams, M. D., & Opar, D. A. (2022). Assessing isometric kicking force and post-match responses using the Kicker test. Journal of sports sciences, 40(11), 1275–1281. https://doi.org/10.1080/02640414.2022.2065772 |
| 1. Burnie, L., Barratt, P., Davids, K., Worsfold, P., & Wheat, J. S. (2022). Effects of strength training on the biomechanics and coordination of short-term maximal cycling. Journal of sports sciences, 40(12), 1315–1324. https://doi.org/10.1080/02640414.2022.2080159 |
| 1. Lambert, C., Riesterer, J., Mauch, M., Lambert, M., Paul, J., & Ritzmann, R. (2022). Modified defense reaction reduces biomechanical and myoelectrical ACL injury risk factors in elite Judo. Journal of sports sciences, 40(12), 1325–1335. https://doi.org/10.1080/02640414.2022.2080160 |
| 1. Slegers, N., & Love, D. (2022). The role of ball backspin alignment and variability in basketball shooting accuracy. Journal of sports sciences, 40(12), 1360–1368. https://doi.org/10.1080/02640414.2022.2080164 |
| 1. Meechan, D., McErlain-Naylor, S. A., McMahon, J. J., Suchomel, T. J., & Comfort, P. (2022). Comparing biomechanical time series data across countermovement shrug loads. Journal of sports sciences, 40(15), 1658–1667. https://doi.org/10.1080/02640414.2022.2091351 |
| 1. Dos'Santos, T., Cowling, I., Challoner, M., Barry, T., & Caldbeck, P. (2022). What are the significant turning demands of match play of an English Premier League soccer team?. Journal of sports sciences, 40(15), 1750–1759. https://doi.org/10.1080/02640414.2022.2109355 |
| 1. Taylor, H. L., Garabello, G., Pugh, J., Morton, J., Langan-Evans, C., Louis, J., Borgersen, R., & Areta, J. L. (2022). Patterns of energy availability of free-living athletes display day-to-day variability that is not reflected in laboratory-based protocols: Insights from elite male road cyclists. Journal of sports sciences, 40(16), 1849–1856. https://doi.org/10.1080/02640414.2022.2115676 |
| 1. Leo, P., Simon, D., Hovorka, M., Lawley, J., & Mujika, I. (2022). Elite versus non-elite cyclist - Stepping up to the international/elite ranks from U23 cycling. Journal of sports sciences, 40(16), 1874–1884. https://doi.org/10.1080/02640414.2022.2117394 |
| 1. Blythman, R., Saxena, M., Tierney, G. J., Richter, C., Smolic, A., & Simms, C. (2022). Assessment of deep learning pose estimates for sports collision tracking. Journal of sports sciences, 40(17), 1885–1900. https://doi.org/10.1080/02640414.2022.2117474 |
| 1. Pitt, J. P., Bracken, R. M., Scott, S. N., Fontana, F. Y., Skroce, K., & McCarthy, O. (2022). Nutritional intake when cycling under racing and training conditions in professional male cyclists with type 1 diabetes. Journal of sports sciences, 40(17), 1912–1918. https://doi.org/10.1080/02640414.2022.2118944 |
| 1. Navandar, A., Kipp, K., & Navarro, E. (2022). Hip and knee joint angle patterns and kicking velocity in female and male professional soccer players: A principal component analysis of waveforms approach. Journal of sports sciences, 40(17), 1919–1930. https://doi.org/10.1080/02640414.2022.2121022 |
| 1. Teune, B., Woods, C., Sweeting, A., Inness, M., & Robertson, S. (2022). The influence of individual, task and environmental constraint interaction on skilled behaviour in Australian Football training. Journal of sports sciences, 40(17), 1991–1999. https://doi.org/10.1080/02640414.2022.2124013 |
| 1. Richardson, R. T., Lerch, B., & Nicholson, K. F. (2022). Evaluation of approaches to estimate scapular kinematics during baseball pitching. Journal of sports sciences, 40(18), 2062–2071. https://doi.org/10.1080/02640414.2022.2133391 |
| 1. Fleeton, J. R. M., Sanders, R. H., & Fornusek, C. (2022). Impact of maximal strength training on countermovement jump phase characteristics in athletes with cerebral palsy. Journal of sports sciences, 40(19), 2118–2127. https://doi.org/10.1080/02640414.2022.2137303 |
| 1. Ross, G. B., Clouthier, A. L., Boyle, A., Fischer, S. L., & Graham, R. B. (2022). Comparison of machine learning classifiers for differentiating level and sport using movement data. Journal of sports sciences, 40(19), 2166–2172. https://doi.org/10.1080/02640414.2022.2145430 |
| 1. Singh, H., Shih, H. T., Kal, E., Bennett, T., & Wulf, G. (2022). A distal external focus of attention facilitates compensatory coordination of body parts. Journal of sports sciences, 40(20), 2282–2291. https://doi.org/10.1080/02640414.2022.2150419 |
| 1. Zanin, M., Azzalini, A., Ranaweera, J., Till, K., Darrall-Jones, J., & Roe, G. (2022). Designing a small-sided game to elicit attacking tactical behaviour in professional rugby union forwards. Journal of sports sciences, 40(20), 2304–2314. https://doi.org/10.1080/02640414.2022.2156101 |
| 1. Clark, J. D., Mallett, C. J., Moyle, G. M., & Coulter, T. J. (2022). Competitive Situations Requiring Mental Toughness in Women's Australian Rules Football. Journal of sports sciences, 40(21), 2412–2423. https://doi.org/10.1080/02640414.2022.2162239 |
| 1. Davis, L., Jowett, S., Sörman, D., & Ekelund, R. (2022). The role of quality relationships and communication strategies for the fulfilment of secure and insecure athletes' basic psychological needs. Journal of sports sciences, 40(21), 2424–2436. https://doi.org/10.1080/02640414.2022.2162240 |
| 1. Moya-Ramón, M., Haakonssen, E., Peña-González, I., Mateo-March, M., & Javaloyes, A. (2022). Predicting BMX Performance with Laboratory Measurements in Elite Riders. Journal of sports sciences, 40(21), 2461–2467. https://doi.org/10.1080/02640414.2022.2162755 |
| 1. Fornasiero, A., Savoldelli, A., Zignoli, A., Callovini, A., Decet, M., Bortolan, L., Schena, F., & Pellegrini, B. (2022). Eager to set a record in a vertical race? Test your VO2max first!. Journal of sports sciences, 40(22), 2544–2551. https://doi.org/10.1080/02640414.2023.2172801 |
| 1. Lam, H. K. N., Sproule, J., Turner, A. P., Murgatroyd, P., Gristwood, G., Richards, H., & Phillips, S. M. (2022). International orienteering experts' consensus on the definition, development, cause, impact and methods to reduce mental fatigue in orienteering: A Delphi study. Journal of sports sciences, 40(23), 2595–2607. https://doi.org/10.1080/02640414.2023.2177027 |
| 1. Jonsson Kårström, M., Staunton, C., McGawley, K., Björklund, G., & Laaksonen, M. S. (2022). Rifle carriage affects gear distribution during on-snow skiing in female and male biathletes. Journal of sports sciences, 40(24), 2722–2731. https://doi.org/10.1080/02640414.2023.2189796 |
| 1. Mason, J., Stewart, A., Kniewasser, C., & Zech, A. (2022). Tracking sleep in the field: sleep quality and sleep behaviours of elite track and field athletes during preparation and competition. Journal of sports sciences, 40(24), 2741–2749. https://doi.org/10.1080/02640414.2023.2191089 |
| 1. Peek, R. J., Carey, D. L., Middleton, K. J., Gastin, P. B., & Clarke, A. C. (2022). Peak movement and impact characteristics of different training methods in professional rugby union. Journal of sports sciences, 40(24), 2760–2767. https://doi.org/10.1080/02640414.2023.2192555 |
| 1. Fransen, K., Boen, F., Haslam, S. A., McLaren, C. D., Mertens, N., Steffens, N. K., & Bruner, M. W. (2022). Unlocking the power of 'us': Longitudinal evidence that identity leadership predicts team functioning and athlete well-being. Journal of sports sciences, 40(24), 2768–2783. https://doi.org/10.1080/02640414.2023.2193005 |
| 1. Sado, N., Yoshioka, S., & Fukashiro, S. (2022). Curved Approach in High Jump Induces Greater Jumping Height without Greater Joint Kinetic Exertions than Straight Approach. Medicine and science in sports and exercise, 54(1), 120–128. https://doi.org/10.1249/MSS.0000000000002761 |
| 1. Rowe, J. T., King, R. F. G. J., King, A. J., Morrison, D. J., Preston, T., Wilson, O. J., & O'Hara, J. P. (2022). Glucose and Fructose Hydrogel Enhances Running Performance, Exogenous Carbohydrate Oxidation, and Gastrointestinal Tolerance. Medicine and science in sports and exercise, 54(1), 129–140. https://doi.org/10.1249/MSS.0000000000002764 |
| 1. Buckley, T. A., Munkasy, B. A., Evans, K. M., & Clouse, B. (2022). Acute Physical and Mental Activity Influence on Concussion Recovery. Medicine and science in sports and exercise, 54(2), 307–312. https://doi.org/10.1249/MSS.0000000000002787 |
| 1. Ito, S., Nakagawa, K., Nakajima, T., Iteya, M., Crawshaw, L., & Kanosue, K. (2022). Dynamic Control of Upper Limb Stretch Reflex in Wrestlers. Medicine and science in sports and exercise, 54(2), 313–320. https://doi.org/10.1249/MSS.0000000000002799 |
| 1. Opar, D. A., Ruddy, J. D., Williams, M. D., Maniar, N., Hickey, J. T., Bourne, M. N., Pizzari, T., & Timmins, R. G. (2022). Screening Hamstring Injury Risk Factors Multiple Times in a Season Does Not Improve the Identification of Future Injury Risk. Medicine and science in sports and exercise, 54(2), 321–329. https://doi.org/10.1249/MSS.0000000000002782 |
| 1. Rockwell, M. S., Kostelnik, S. B., McMillan, R. P., Lancaster, M., Larson-Meyer, D. E., & Hulver, M. W. (2022). An Association between Bioavailable 25-Hydroxyvitamin D and Bone Mineral Density in a Diverse Cohort of Collegiate Athletes. Medicine and science in sports and exercise, 54(3), 371–376. https://doi.org/10.1249/MSS.0000000000002807 |
| 1. McKay, A. K. A., Peeling, P., Pyne, D. B., Tee, N., Whitfield, J., Sharma, A. P., Heikura, I. A., & Burke, L. M. (2022). Six Days of Low Carbohydrate, Not Energy Availability, Alters the Iron and Immune Response to Exercise in Elite Athletes. Medicine and science in sports and exercise, 54(3), 377–387. https://doi.org/10.1249/MSS.0000000000002819 |
| 1. Geng, Y. U., Zhang, L., & Wu, X. (2022). Effects of Blood Flow Restriction Training on Blood Perfusion and Work Ability of Muscles in Elite Para-alpine Skiers. Medicine and science in sports and exercise, 54(3), 489–496. https://doi.org/10.1249/MSS.0000000000002805 |
| 1. Rasica, L., Inglis, E. C., Iannetta, D., Soares, R. N., & Murias, J. M. (2022). Fitness Level- and Sex-Related Differences in Macrovascular and Microvascular Responses during Reactive Hyperemia. Medicine and science in sports and exercise, 54(3), 497–506. https://doi.org/10.1249/MSS.0000000000002806 |
| 1. Coates, A. M., Cheung, C. P., Currie, K. D., King, T. J., Mountjoy, M. L., & Burr, J. F. (2022). Differences in Left Ventricular Function at Rest and during Isometric Handgrip Exercise in Elite Aquatic Sport Athletes. Medicine and science in sports and exercise, 54(3), 507–516. https://doi.org/10.1249/MSS.0000000000002812 |
| 1. Facer-Childs, E. R., Mascaro, L., Hoffman, D., Mansfield, D., Drummond, S. P. A., & Rajaratnam, S. M. W. (2022). Sleep as a Major Determinant for Mental Health Outcomes in Elite Australian Football League (AFL) Athletes. Medicine and science in sports and exercise, 54(4), 665–672. https://doi.org/10.1249/MSS.0000000000002825 |
| 1. Morehen, J. C., Rosimus, C., Cavanagh, B. P., Hambly, C., Speakman, J. R., Elliott-Sale, K. J., Hannon, M. P., & Morton, J. P. (2022). Energy Expenditure of Female International Standard Soccer Players: A Doubly Labeled Water Investigation. Medicine and science in sports and exercise, 54(5), 769–779. https://doi.org/10.1249/MSS.0000000000002850 |
| 1. Hajek, M., Williams, M. D., Bourne, M. N., Roberts, L. A., Morris, N. R., Shield, A. J., Mingin, C. V., Headrick, J., & Duhig, S. J. (2022). Predicting Noncontact Lower Limb Injury Using Lumbar Morphology in Professional Australian Football and Rugby League Players. Medicine and science in sports and exercise, 54(5), 814–820. https://doi.org/10.1249/MSS.0000000000002847 |
| 1. Blauwet, C. A., Chakraverty, J., Derman, W., Idrisova, G., Martin, P., Miller, S. C., Morrissey, D., & Webborn, N. (2022). Shoulder Pain, Function, and Ultrasound-Determined Structure in Elite Wheelchair-Using Para Athletes: An Observational Study. Medicine and science in sports and exercise, 54(6), 896–904. https://doi.org/10.1249/MSS.0000000000002875 |
| 1. Stemper, B. D., Harezlak, J., Shah, A. S., Rowson, S., Mihalik, J. P., Riggen, L., Duma, S., Pasquina, P., Broglio, S. P., McAllister, T. W., McCrea, M. A., & CARE Consortium Investigators (2022). Association between Preseason/Regular Season Head Impact Exposure and Concussion Incidence in NCAA Football. Medicine and science in sports and exercise, 54(6), 912–922. https://doi.org/10.1249/MSS.0000000000002874 |
| 1. Kakehata, G., Goto, Y., Iso, S., & Kanosue, K. (2022). The Timing of Thigh Muscle Activity Is a Factor Limiting Performance in the Deceleration Phase of the 100-m Dash. Medicine and science in sports and exercise, 54(6), 1002–1012. https://doi.org/10.1249/MSS.0000000000002876 |
| 1. Ainegren, M., Linnamo, V., & Lindinger, S. (2022). Effects of Aerodynamic Drag and Drafting on Propulsive Force and Oxygen Consumption in Double Poling Cross-Country Skiing. Medicine and science in sports and exercise, 54(7), 1058–1065. https://doi.org/10.1249/MSS.0000000000002885 |
| 1. Collings, T. J., Diamond, L. E., Barrett, R. S., Timmins, R. G., Hickey, J. T., DU Moulin, W. S., Williams, M. D., Beerworth, K. A., & Bourne, M. N. (2022). Strength and Biomechanical Risk Factors for Noncontact ACL Injury in Elite Female Footballers: A Prospective Study. Medicine and science in sports and exercise, 54(8), 1242–1251. https://doi.org/10.1249/MSS.0000000000002908 |
| 1. Wille, C. M., Stiffler-Joachim, M. R., Kliethermes, S. A., Sanfilippo, J. L., Tanaka, C. S., & Heiderscheit, B. C. (2022). Preseason Eccentric Strength Is Not Associated with Hamstring Strain Injury: A Prospective Study in Collegiate Athletes. Medicine and science in sports and exercise, 54(8), 1271–1277. https://doi.org/10.1249/MSS.0000000000002913 |
| 1. Freire, R., Pereira, G. R., Alcantara, J. M. A., Santos, R., Hausen, M., & Itaborahy, A. (2022). New Predictive Resting Metabolic Rate Equations for High-Level Athletes: A Cross-Validation Study. Medicine and science in sports and exercise, 54(8), 1335–1345. https://doi.org/10.1249/MSS.0000000000002926 |
| 1. Schmida, E. A., Wille, C. M., Stiffler-Joachim, M. R., Kliethermes, S. A., & Heiderscheit, B. C. (2022). Vertical Loading Rate Is Not Associated with Running Injury, Regardless of Calculation Method. Medicine and science in sports and exercise, 54(8), 1382–1388. https://doi.org/10.1249/MSS.0000000000002917 |
| 1. Aune, S. K., Bonnevie-Svendsen, M., Nyborg, C., Trøseid, M., Seljeflot, I., Hisdal, J., & Helseth, R. (2022). Gut Leakage and Cardiac Biomarkers after Prolonged Strenuous Exercise. Medicine and science in sports and exercise, 54(9), 1476–1482. https://doi.org/10.1249/MSS.0000000000002948 |
| 1. Rønnestad, B. R., Urianstad, T., Hamarsland, H., Hansen, J., Nygaard, H., Ellefsen, S., Hammarström, D., & Lundby, C. (2022). Heat Training Efficiently Increases and Maintains Hemoglobin Mass and Temperate Endurance Performance in Elite Cyclists. Medicine and science in sports and exercise, 54(9), 1515–1526. https://doi.org/10.1249/MSS.0000000000002928 |
| 1. Tso, J. V., Liu, C., Turner, C. G., Uppal, K., Prabakaran, G., Ejaz, K., Baggish, A. L., Jones, D. P., Quyyumi, A. A., & Kim, J. H. (2022). Metabolic Alterations Differentiating Cardiovascular Maladaptation from Athletic Training in American-Style Football Athletes. Medicine and science in sports and exercise, 54(10), 1617–1624. https://doi.org/10.1249/MSS.0000000000002960 |
| 1. Tiller, N. B., Wheatley-Guy, C. M., Fermoyle, C. C., Robach, P., Ziegler, B., Gavet, A., Schwartz, J. C., Taylor, B. J., Constantini, K., Murdock, R., Johnson, B. D., & Stewart, G. M. (2022). Sex-Specific Physiological Responses to Ultramarathon. Medicine and science in sports and exercise, 54(10), 1647–1656. https://doi.org/10.1249/MSS.0000000000002962 |
| 1. Warden, S. J., Sventeckis, A. M., Surowiec, R. K., & Fuchs, R. K. (2022). Enhanced Bone Size, Microarchitecture, and Strength in Female Runners with a History of Playing Multidirectional Sports. Medicine and science in sports and exercise, 54(12), 2020–2030. https://doi.org/10.1249/MSS.0000000000003016 |
| 1. Miller, R., Balshaw, T. G., Massey, G. J., Maeo, S., Lanza, M. B., Haug, B., Johnston, M., Allen, S. J., & Folland, J. P. (2022). The Muscle Morphology of Elite Female Sprint Running. Medicine and science in sports and exercise, 54(12), 2138–2148. https://doi.org/10.1249/MSS.0000000000002999 |
| 1. Whales, L., Frawley, S., Cohen, A., & Nikolova, N. (2022) We are a team of leaders: practicing leadership in professional sport, Sport Management Review, 25:3, 476-500, |
| 1. Habeeb, C., Warner, S., & Walsh, D. (2022) Managing mental health: athlete help-seeking, Sport Management Review, 25:5, 871-891, |
